# Why Brain Criticality Is Clinically Relevant: A Scoping Review

**DOI:** 10.3389/fncir.2020.00054

**Published:** 2020-08-26

**Authors:** Vincent Zimmern

**Affiliations:** Division of Child Neurology, The University of Texas Southwestern Medical Center, Dallas, TX, United States

**Keywords:** criticality, brain, long-range temporal correlation, neurodevelopment, neurodegeneration, sleep, epilepsy, anesthesia

## Abstract

The past 25 years have seen a strong increase in the number of publications related to criticality in different areas of neuroscience. The potential of criticality to explain various brain properties, including optimal information processing, has made it an increasingly exciting area of investigation for neuroscientists. Recent reviews on this topic, sometimes termed brain criticality, make brief mention of clinical applications of these findings to several neurological disorders such as epilepsy, neurodegenerative disease, and neonatal hypoxia. Other clinicallyrelevant domains – including anesthesia, sleep medicine, developmental-behavioral pediatrics, and psychiatry – are seldom discussed in review papers of brain criticality. Thorough assessments of these application areas and their relevance for clinicians have also yet to be published. In this scoping review, studies of brain criticality involving human data of all ages are evaluated for their current and future clinical relevance. To make the results of these studies understandable to a more clinical audience, a review of the key concepts behind criticality (e.g., phase transitions, long-range temporal correlation, self-organized criticality, power laws, branching processes) precedes the discussion of human clinical studies. Open questions and forthcoming areas of investigation are also considered.

## Introduction

The brain criticality hypothesis suggests that neural networks and thus, many aspects of brain activity self-organize into a critical state (Wilting and Priesemann, 2019). Critical states are unique configurations of physical systems that have been a central focus of statistical physics for more than a century. Criticality, which is a synonymous term for “critical phenomena” or “critical states,” marks the transition between ordered and disordered states. The theory of critical phenomena has found applications in many scientific fields, including neuroscience and clinical neurology (Sornette, 2004; Cocchi et al., 2017). In the neurosciences, criticality is appealing because theory and modeling suggest that neural networks at criticality exhibit optimal processing and computing properties (Beggs, 2008; Shew and Plenz, 2013). These properties include information transmission, information storage, dynamic range, metastable states, and computational power (Maass et al., 2002; Bertschinger and Natschläger, 2004; Latham and Nirenberg, 2004; Haldeman and Beggs, 2005; Kinouchi and Copelli, 2006; Tanaka et al., 2009; Boedecker et al., 2012; Shriki et al., 2013; Gautam et al., 2015; Shriki and Yellin, 2016; Hoffmann and Payton, 2018). The term “brain criticality” is used in this review as a catch-all term for the various manifestations of critical phenomena in human neuroscience.

**FIGURE 1:**
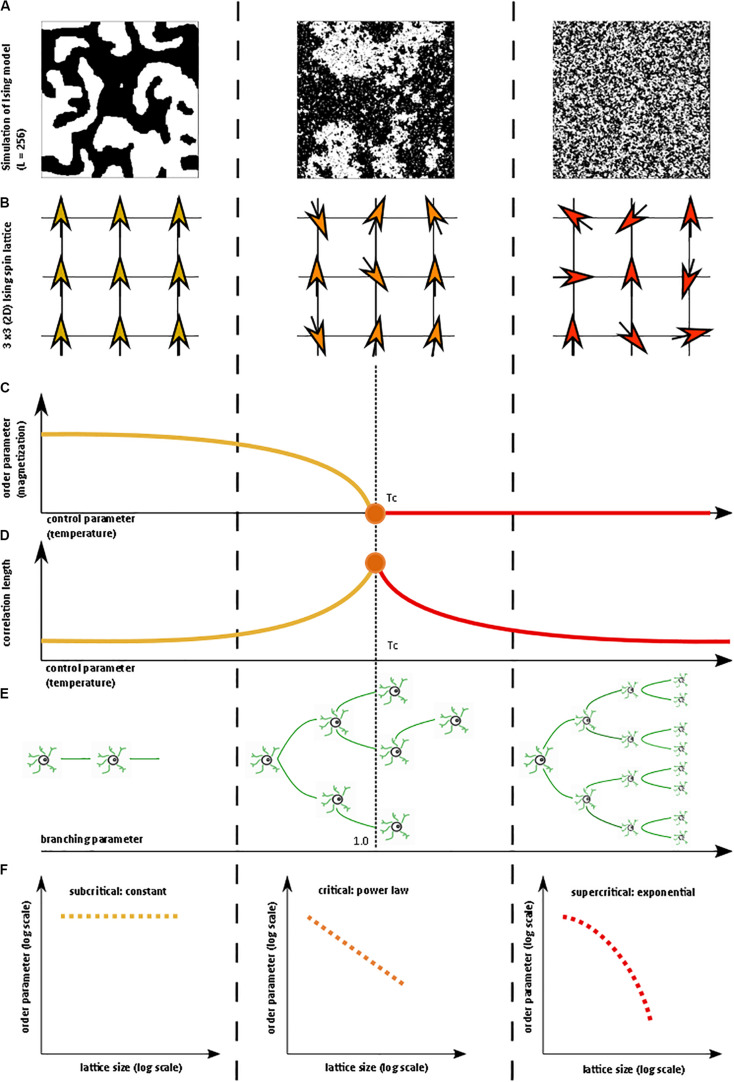
Illustration of critical phase transition using the two-dimensional (2D) Ising model. For more information on the Ising model, see Nishimori and Ortiz (2011), Beggs and Timme (2012). **(A)** Simulation of 2D Ising model with length = 256 in subcritical, critical, and super-critical states as temperature increases from left to right. Black and white areas represent magnetization domains with differing spins. Simulations were generated using open-source code from Matt Bierbaum (mattbierbaum.github.io/ising.js) using the Metropolis algorithm. **(B)** Illustration of 2D Ising spin lattice to show differing spin states during phase transition. Image adapted with permission from Beggs and Timme (2012). The organized spins in the subcritical state give way to random spin arrangements in the supercritical state, passing through an intermediate critical state with a complex arrangement of spins. **(C)** The order parameter of the system decreases smoothly as the control parameter increases, until it abruptly changes at the critical temperature (Tc or the Curie temperature). **(D)** The correlation length is maximized at the critical point. **(E)** A branching parameter of 1 allows an aggregate one-to-one transmission of neural signals. Branching parameter greater than 1 leads to supercritical, run-away neuronal excitation. Branching parameter less than 1 leads to subcritical dying-off of the neuronal transmission. **(F)** At the critical point, observables of the system, including the order parameter, obey a power-law or scale-free distribution, seen as a straight-line on a log-log plot. The order parameter remains constant in the subcritical case, while it drops off exponentially in the supercritical state.

This scoping review of the brain criticality literature will start with a section that reviews the physics-based ideas behind brain criticality, including a discussion of the Ising model. It will then review the brain criticality literature in each of seven domains (i.e., anesthesia, epilepsy, neurodegeneration, neurodevelopment, cognition, sleep medicine, and psychiatry), focusing on the clinical applications. This review should serve as an entry-point for clinicians and translational researchers interested in using this conceptual framework and its associated tools to advance patient care. Understandably, it is not possible to summarize nearly a century of developments in statistical physics − not to mention 30 years of applications of these physical concepts to neuroscience−in a single review paper. An excellent introductory paper for newcomers to criticality is Beggs and Timme, 2012. References are available for those interested in diving into the technical details of critical phenomena (Nishimori and Ortiz, 2011; Tomko et al., 2018), including two books on criticality in neural dynamics (Plenz and Niebur, 2014; Tomen et al., 2019). In this section, criticality is introduced from the perspective of phase transitions and is illustrated using the two-dimensional Ising model (see Figure 1). This is followed by a discussion of the various characteristics of critical phenomena and a brief discussion of self-organized criticality.

## Criticality and Associated Concepts

In statistical physics, criticality refers to the behavior that is seen when a physical system undergoes a specific kind of phase transition. Typically, a macroscopic property of the system called the *order parameter* changes as a function of an underlying feature called the *control parameter*. In the example of a vapor-to-water phase transition, the order parameter is the macroscopic appearance reflective of the phase’s entropy (i.e., water or vapor), and the control parameter is the temperature (Hesse and Gross, 2014). Generally, gradual changes in the control parameter lead to similarly gradual changes in the order parameter. However, at specific points, the order parameter changes abruptly. On a plot of the order parameter on the *y*-axis and the control parameter on the *x*-axis (i.e., a phase diagram), there is either a jump (i.e., a discontinuity) or a sharp corner (i.e., a non-differentiable point) at the transition point of a phase transition. If the change in the order parameter is a jump, the phase transition is known as first-order or discontinuous. If the change in the order parameter is a sharp corner, the phase transition is known as second-order or continuous. The second-order phase transition allows a system to be at the exact transition point at the interface between two very different states, with usually one state being more disordered than the other. The system is said to be “at criticality” or in the critical state (Nishimori and Ortiz, 2011; Hesse and Gross, 2014). The phase in which the control parameter is below the critical value is called the subcritical phase, while the phase in which the control parameter is above the critical value is called the supercritical phase. It is important to note that the theory of phase transitions usually involves systems at the thermodynamic limit, i.e. infinitelylarge volume. When not at the thermodynamic limit, phase transitions occur over a parameter range called a Griffiths phase rather than at a single critical point and are sometimes referred to as quasi-critical states (Moretti and Munoz, 2013). Quasi-critical states obey many of the properties of a critical system but are not entirely critical.

These concepts can be illustrated with the help of the two-dimensional Ising model, which is a classic example of a critical transition in ferromagnetism (Beggs and Timme, 2012). The Ising model consists of a lattice in a piece of iron, with each site of the lattice corresponding to a dipole moment (i.e., an up or down spin) (Figure 1B). Each dipole moment operates like a bar magnet and can influence its nearest neighbors to align in the same direction. At a low temperature, nearest-neighbor effects will dominate the system. At a fixed temperature below the critical temperature, a cluster of aligned spins will get larger and larger, and, with time, will take over the entire lattice to make a uniform dipole moment (Figure 1A). Thus, at this low temperature, the piece of iron will behave like a magnet because the spins will align throughout the lattice to yield a strong net magnetization (i.e., order parameter). However, as temperature (i.e., control parameter) increases, the energy from heat begins to jostle the spins. Past a critical temperature called the Curie temperature (Tc), the disordered spins from the added heat will overwhelm the ordering effect of the nearest-neighbor interaction, leading to a loss of the magnetization (Figures 1A–C). Heat, therefore, takes the system from a subcritical, magnetic phase through a critical phase transition and on to a supercritical, non-magnetic and disordered phase. At the critical temperature, a critical phase emerges where order and disorder are evenly matched (Nishimori and Ortiz, 2011; Beggs and Timme, 2012). The correlation length (i.e., how far a single spin change can propagate through the system) is maximized in this phase (Figure 1D), and in the infinitely large system, goes to infinity at the critical point. The order parameter (i.e., magnetization)−along with other observables like magnetization domain size and magnetic susceptibility−become power-law distributed with unique power-law exponents (Figure 1F). Power laws refer to a probability density function of the form of *p*(*x*) = *Cx*^−α^ for some *x* > *x_o* and with αcorresponding to the power-law exponent. Power laws exhibit scale invariance and are therefore called scale-free. A function *f*(*x*) is scale invariant if *f*(*cx*)∝*f*(*x*), where α signifies “proportional to.” In other words, scaling the argument of the function is equivalent to a proportional scaling of the function itself. In the case of the power-law, *f*(*cx*) = (*cx*)^−α^ = c^−α^ x^−α^ =c^−α^ f(x) ∝*f*(*x*). Moreover, because *log*⁡(*f*(*x*)) = log∝(*x*^−α^) = −α*log*⁡(*x*), a log-log plot of a power-law distributed dataset should produce a straight line with slope −α. Caveats on using this log-log plot technique to extract the power-law exponent are addressed in a subsequent section.

The Ising model can lead to very complex behavior patterns and has been used to model neural networks (Fraiman et al., 2009; Beggs and Timme, 2012; Deco et al., 2012; Marinazzo et al., 2013; Stramaglia et al., 2017). The Ising model and several other well-characterized models have led to a better understanding of how critical systems behave. Nevertheless, critical systems remain difficult to identify because the relevant order and control parameters may not always be readily available for experimentation, making it difficult to construct a complete phase diagram. In the absence of a complete phase diagram, one can use several known markers of criticality, as follows:

### Branching Parameter

A branching parameter σ, in the setting of brain criticality, is the ratio of downstream activated neurons to upstream activated neurons (Harris, 1963). In other words, as in Figure 1E, a branching parameter of 1 means that every activated neuron on average fires or activates one other downstream neuron (Hobbs et al., 2010). Branching parameter less than 1 indicates a subcritical phase that evolves with time to a quiescent, inactive state. On the other hand, a branching parameter greater than 1 indicates a supercritical phase of increasing activity. Of course, branching parameters are variable and dynamic. Caution is needed in the interpretation of the branching parameter, however, because a branching parameter of 1 can also be observed in certain supercritical states (Hesse and Gross, 2014).

### Long-Range Temporal Correlation, Critical Slowing, and Flickering

**FIGURE 2:**
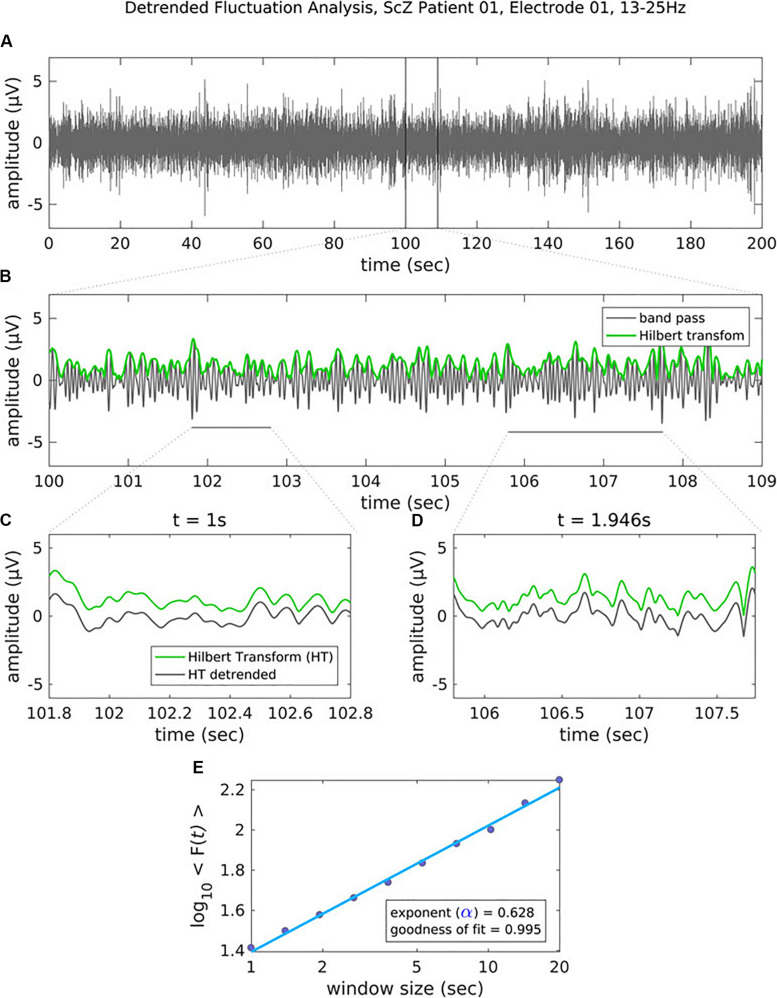
Figure legend taken from Moran et al. (2019) and reproduced with permission. This figure depicts the steps of the detrended fluctuation analysis (DFA). **(A)** 200 s EEG recording that has been band-pass filtered (13–25 Hz, channel 01 = Cz) in a patient with schizophrenia. **(B)** A close-up view (9 s) of the band-pass filtered EEG along with the amplitude envelope derived from the Hilbert transformation (in green), which is used for estimating the scaling exponent. **(C–D)** Two examples of the amplitude envelope with two window sizes. In the left graph, no detrending (i.e., removal of the trend line) has been applied whereas in the right graph, detrending has been applied. For each time window size, the fluctuation of the detrended signal is calculated as the mean standard deviation over all identical sized segments **(E)** The DFA scaling exponent is given by the slope of the log-log plot between fluctuation F and window size. A DFA scaling exponent between 0.5 and 1 indicates the presence of LRTC – in this case, the correlation extends up to 20 s. In this example, the DFA scaling exponent for the beta-band-oscillations in this channel is α = 0.628. It is common to report the R^2^ value of the linear regression of the log-log plot as a goodness-of-fit score.

In critical systems, the response of the system to external stimuli – called the dynamic range or dynamic correlation – is maximized. Small perturbations of the system at criticality lead to geometric (rather than exponential) returns to the steady-state (Hesse and Gross, 2014), leading to *long-range temporal correlation* (LRTC or long-memory). One can measure LRTC in multiple ways. Popular methods include the Hurst exponent (through various estimators) and detrended fluctuation analysis (DFA) (Peng et al., 1995a, b; Simonsen et al., 1998; Hardstone et al., 2012). DFA produces a *scaling exponent* over a defined time period (see Figure 2 for an illustration of DFA). If that scaling exponent is between 0.5 and 1, with a good fit (see Figure 2), one can conclude that the time series exhibits LRTC over that time period.

This geometric rate of return to steady-state is also called *critical slowing down* (Scheffer et al., 2009; Van De Leemput et al., 2014). More generally, at the critical point, the dynamic correlation of the system diverges such that avalanches (i.e., network activity) occur at all scales of the system (Hesse and Gross, 2014). Another phenomenon seen at the critical transition is called *flickering*, which emerges when noise allows a system to migrate back-and-forth between two attractor basins (Wang et al., 2012). This phenomenon is not to be confused with flicker noise or *1/f* noise, which is discussed next.

### The Emergence of Power-Law (1/*f*) Noise and Power-Law Observables

**FIGURE 3:**
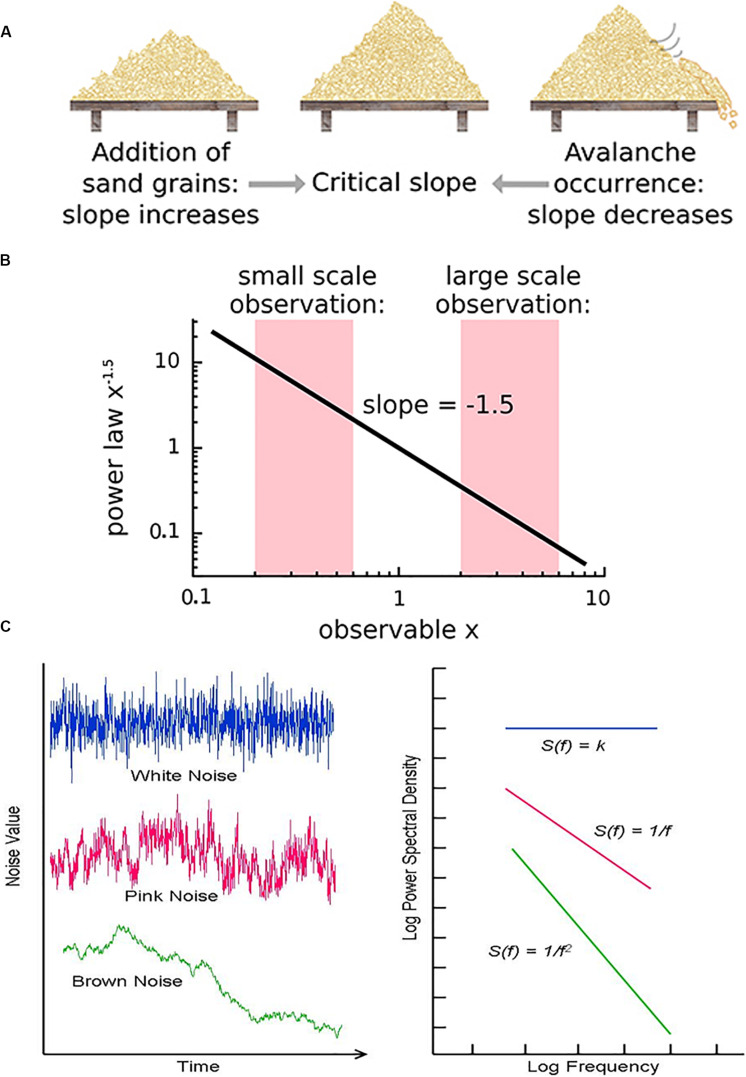
**(A)** The sandpile model is a classic model of self-organized criticality (SOC), derived as a though experiment (Bak et al., 1988) and proven later with rice piles (Frette et al., 1996). Sand is dropped continuously at a fixed rate onto a flat surface. A sandpile forms. As the sand is added, the slope of the pile increases and avalanches of sand grains begin to occur. If the slope exceeds a “critical slope,” small and large avalanches will decrease the slope back closer to the critical slope. **(B)** At the critical slope, the distribution of avalanche sizes (i.e., number of sand grains) is power-law distributed. On a log-log plot, the slope of a power-law-distributed variable gives the power-law exponent. Most avalanches are small but a non-negligible number are quite large, up to nearly the size of the entire system. **(A,B)** reused with permission from Hesse and Gross (2014). **(C)** Neurophysiologic measurements such as voltage from an EEG recording can be displayed as time series that exhibit characteristic spectral densities S(*f*). **(C)** Figure reused with permission from Scholarpedia and E. Izhikevich. Plot of different color-coded time series (left) and a log-log plot of their respective spectral densities (right). In Gaussian “white” noise, each frequency has equal energy, leading to a constant spectral density. “Pink” noise, which is characteristic of SOC but arises in many other settings, has a spectral density given by 1/f, thus the name “1/f noise.” “Brown” noise refers to Brownian or random motion, whose spectral density is typically given by 1/f^2^.

Critical systems, when perturbed by weak inputs, will exhibit superposed geometric responses to the inputs, which yields *1/f* noise, also called pink noise, power-law noise, or flicker noise (see Figure 3C). Many authors employ these terms synonymously with long-range dependence or long-memory, as these are identical phenomena. The term *1/f* noise refers to the phenomenon in which the power spectrum *S*(*f*) of a time series obeys a power law of the form*S*(*f*) = α*f*^−β^. Historically, the cases of β = 0,β = 1,β = 2 are referred to as “white” noise, “pink” noise, and “brown” noise, respectively (Li et al., 2005). The range 0.5 < β < 1.5 is commonly accepted as *1/f* noise. While all critical systems should exhibit *1/f* noise, not all *1/f* noise is indicative of criticality (Bédard et al., 2006; Hesse and Gross, 2014).

As mentioned earlier, multiple observables will follow a power-law distribution when in a critical state. However, power laws are necessary but not sufficient to prove the existence of criticality – many other processes can generate a power-law distribution. For more on this topic, see the following references (Mitzenmacher, 2004; Newman, 2005; Touboul and Destexhe, 2010). Demonstrating the existence of a power law is not straightforward. As a rule, power laws take the form of *Y* = *Cx*^−α^ for some *x* > *x*_*o*_. For many years, it was common practice to plot *y* against *x* (from the previous formula) in log-log coordinates – if a straight line emerged, the slope of that line was interpreted as the power-law exponent α (see Figure 3B). This approach led to many false claims of power-laws since many other finite datasets (e.g., from a log-normal distribution) can also approximate a straight line on a log-log plot. Since Clauset et al. (2009) and subsequent work, researchers have had access to more sophisticated statistical methodology to argue that their variable obeys a power law, as opposed to other heavy-tailed distributions. While definitions vary, heavy-tail distributions are probability distributions whose tails are “heavier” than the exponential distribution, of which the Gaussian is a sub-type. Examples include the Fisher-Tippett (double-exponential) distribution, the log-normal distribution, the Weibull distribution, among many others. An exhaustive review of several heavy-tailed distributions and their role in neuroscience can be found in Roberts et al. (2015). In this review, publications that claim a power-law distribution based on current practices are contrasted with publications that rely on the previously accepted “log-log approach.”

### Relationship of Power-Law Exponents to Each Other

In systems at criticality, many observable variables (e.g., correlation, size distribution) obey power laws. The different exponents of these power laws are inter-related. The details of these mathematical relationships are beyond the scope of this review but represent a fascinating topic in their own right. To give but one example, avalanche size distribution with power exponent α and avalanche lifetime distribution with power exponent β should be related to avalanche lifetime γ by the following equation: $\gamma =\frac{\beta -1}{\alpha -1}$. This relationship has been experimentally validated in individual neurons (Beggs and Timme, 2012; Friedman et al., 2012).

### Scaling Function

**FIGURE 4:**
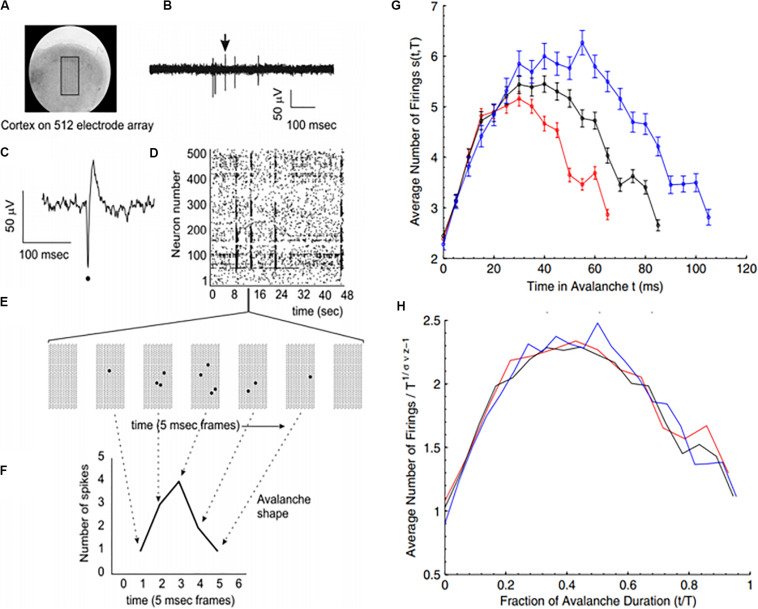
Avalanches recorded from neural tissue. **(A)** Micrograph of a cortical slice on an electrode array, represented by a black rectangle. **(B)** Electrical potential recorded from an electrode. Arrow points to a spike from an individual neuron. The spike is expanded in panel **(C)** and marked by a black dot. **(D)** Raster plot of spike times (dots) from multiple neurons over time. **(E)** Expanding the view of the raster plot reveals an avalanche. Each frame represents the electrode array during a single 5 ms period. Black dots are spikes detected on the array. An avalanche is made up of consecutively active frames, beginning and ending with inactive frames. **(F)** Plotting the number of spikes in each frame versus time produces an avalanche shape. **(G)** Avalanche shapes are produced by averaging the temporal profiles of all avalanches of a particular duration from an experimental dataset close to criticality. Different colors here represent different durations. **(H)** The collapses are plotted by rescaling the horizontal and vertical axes. The tight overlap after rescaling is indicative of criticality. Images reproduced with permission Beggs and Timme (2012) and Friedman et al. (2012).

Because of the self-similar or fractal nature of the avalanches of activity in critical systems, the “shape” of avalanche activity is also expected to behave as a fractal (see Figures 4A–G). Therefore, all cascades of activity at criticality ought to be re-scalable to a unique shape, as a function of time (or duration) and power exponents (see Figure 4H). This phenomenon of critical systems allows a “data collapse” or “shape collapse” of all activity onto a single unique shape (Friedman et al., 2012). In some neural avalanches, this shape takes the form of an inverted parabola (Beggs and Timme, 2012). This kind of shape collapse has been observed in many critical systems (Perković et al., 1995; Mehta et al., 2006; Papanikolaou et al., 2011).

There are various theories as to how physical systems can bring themselves to criticality. In the case of the brain, one of the popular theories is called *self-organized criticality* or *SOC* for short (Bak et al., 1987; Christensen et al., 1992; Hesse and Gross, 2014). It is also referred to as *self-organized quasi-criticality* (Bonachela and Muñoz, 2009) since, as mentioned earlier, true criticality occurs only in infinitelysized systems. Introduced initially by Bak et al. (1987), the idea of SOC is that the control parameter is constantly being adjusted to the critical value by a decentralized feedback mechanism. In other words, the control parameter spontaneously decreases when the system is in a supercritical phase and increases when in a subcritical phase. The use of the term “control parameter” is maintained, even if the parameter is not being controlled externally but rather by the system itself (Hesse and Gross, 2014). Figure 3A illustrates one of the earliest models of SOC, called the sandpile model (Bak et al., 1988; Frette et al., 1996). Imagine that sand is accumulating at a certain rate on a flat surface. As the sandpile rises, the slope of the sandpile increases. At a critical point, the distribution of sand-pile avalanche sizes (i.e., a few grains of sand or the entire pile) obeys a power-law. With a power-law or scale-free distribution, avalanches of all scales can occur, but most avalanches will be small with a few being very large (Figure 3B). As sand continues to be added at the same rate, larger avalanches take place and the slope decreases back to the “critical slope.” The criticality in this system is therefore self-organized and the control parameter does not have to be tuned externally, as it does for example in the Ising model.

The internal “tuning” of SOC models for the brain is reminiscent of the concept of homeostatic plasticity. Homeostatic plasticity refers to the capacity of neurons to regulate their excitability based on network activity (Turrigiano and Nelson, 2004). Criticality seems to be connected to homeostatic plasticity though the exact details remain unclear. A recent experiment, however, has cast some light on this question. Rat cortical networks exhibiting criticality in controlled circadian conditions lost their network criticality when deprived of visual inputs. However, signs of criticality resumed in under 48 h with neuronal firing rates being maximally inhibited. This finding suggests that homeostatic regulation of inhibition plays an important role in generating criticality (Ma et al., 2019).

## Article Selection

**FIGURE 5:**
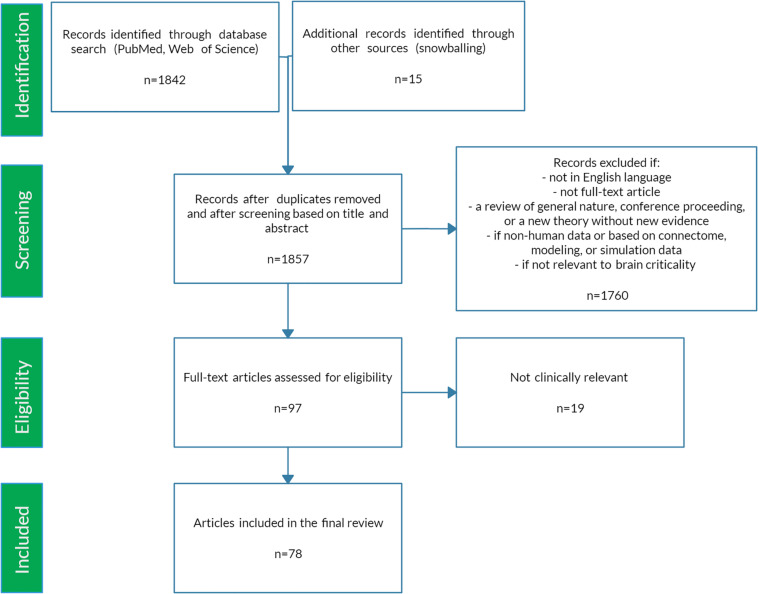
PRISMA-ScR flow chart.

**FIGURE 6:**
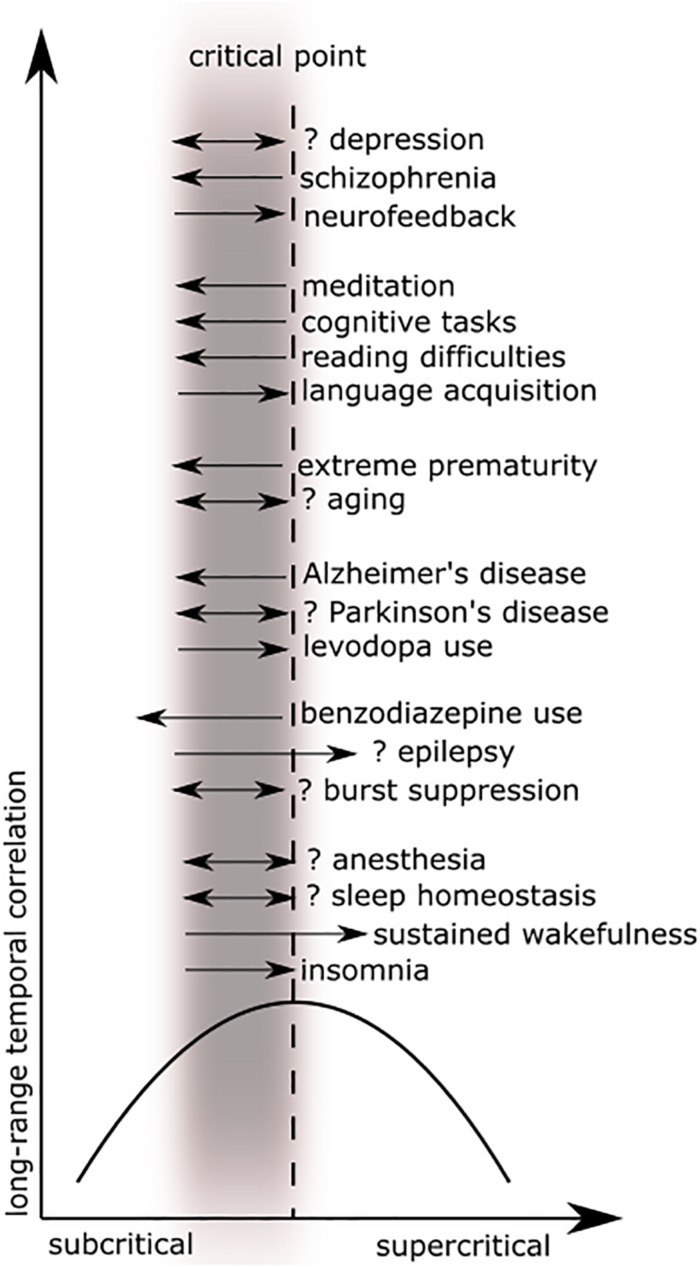
These plots illustrate long-range temporal correlation (LRTC) in different clinical areas as a function of criticality. Figure adapted from Colombo et al. (2016) with permission. The black parabolic curve represents the dynamics of long-range temporal correlation from the subcritical state, reaching a theoretical maximum at the critical point, and decreasing in a supercritical state. The gray blurred rectangle represents the physiological range of brain dynamics, which is thought to be near or below the critical point. Black arrows (excluding the *x*- and *y*-axes) represent deviations toward subcriticality (to the left) or toward supercriticality (to the right) with associated decreases in correlation along the black parabolic curve. Double arrows imply that evidence for both increases and decreases (or absence) in LRTC have been reported in that disease process. See the relevant tables for further detail.

The preceding review was conducted to set the stage for a scoping review of the literature (Colquhoun et al., 2014) using PRISMA-ScR methodology (Moher et al., 2009). A scoping review format was chosen since the nature of this literature is large, heterogeneous, and not amenable to a more precise systematic review. See Figure 5 for a schematic of the article selection process. PubMed and Web of Science databases were searched from their inception until March 2020. The search terms were: “criticality anesthesia,” “criticality brain,” “criticality epilepsy,” “criticality neural,” “criticality neurology,” “criticality seizure,” and “criticality sleep.” A snowball approach identified additional studies that were not captured by the search terms. The reviewer screened titles and abstracts for English language original articles with full-text availability. The following manuscripts were excluded: (1) reviews of a general nature, conference proceedings, and publications offering new theories but without empirical evidence, (2) papers based on non-human data, on connectome data, or mainly on modeling or simulation, (3) duplicates and studies not relevant to brain criticality. The remaining articles were then evaluated for eligibility based on clinical relevance. Ultimately, seventy-eight studies met study criteria for the scoping review. These articles were then analyzed according to seven major categories: anesthesia, epilepsy, neurodegeneration, neurodevelopment, cognition, psychiatry, and sleep medicine. Figure 6 summarizes the changes in LRTC along a subcriticality–supercriticality spectrum that are thought to occur in disease states belonging to these seven clinical categories.

## Clinical Applications of Brain Criticality

### Anesthesia

The literature on criticality in anesthesia is limited but has already shown signs of significant clinical promise. From this small body of research, one can extract two areas of clinical relevance: markers for the depth of anesthesia and predictors of recovery from a persistent comatose state. This literature harmonizes well overall with that of sleep medicine (see Sleep Medicine below).

Three studies have reported on criticality-based markers of the anesthesia depth (see Table 1). An electrocorticogram (ECoG) study of patients undergoing anesthesia for surgical removal of epileptic foci showed that the critical eigenvalues of matrices obtained from a vector auto-regressive (VAR) model could potentially serve as a marker of the depth of anesthesia (Alonso et al., 2014). A scalp electroencephalogram (EEG) study of patients under anesthesia found that LRTC, in combination with oscillation amplitude, could help differentiate between consciousness and anesthesia-induced unconsciousness (Thiery et al., 2018). The authors found that, under anesthesia, LRTC increases in the beta frequency range in frontocentral channels. This increase may reflect decreased neuronal excitability, leading to signal persistence and a resulting limitation on cognitive processes (He, 2014; Thiery et al., 2018). The results of this study contradict a previous study that had found a decrease in LRTC during anesthesia (Krzemiński et al., 2017). However, the contradiction may be secondary to significant differences between the experiments. Thiery et al. (2018) examined human subjects undergoing light anesthesia with sevoflurane, while Krzemiński et al. (2017) examined macaques undergoing deep anesthesia with different anesthetic agents. These differences suggest that future research should focus on identifying criticality-based metrics (e.g., power-law estimation, VAR eigenvalues, LRTC) that are specific to each anesthetic agent at different depths of anesthesia.

**TABLE 1:** Summary of anesthesia-related criticality literature.

Study	Study population	Modality	Analysis	Main findings
Alonso et al., 2014	3 adult subjects with intractable epilepsy undergoing surgical removal of an epileptic focus	ECoG under propofol anesthesia	Vector auto-regressive (VAR) model; critical eigenvalues	• Eigenvalues of VAR matrices change significantly as anesthesia is induced. This finding is robust to changes in how data is normalized and could be used as a metric for depth of anesthesia. • As anesthesia is induced, high frequency modes are damped, suggesting that cognitive processes associated with higher frequencies are being tuned out while lower frequency processes are associated with maintaining the patient alive during anesthesia. • Self-organized criticality (SOC) could be result of synaptic adaptation. Disrupting synaptic adaptation should lead to loss of SOC.
Liu et al., 2014	8 healthy adults receiving propofol infusion; 5 adults with unresponsive wakefulness syndrome (UWS)	fMRI	Power-law estimation	• Node degree distribution was power-law distributed for healthy participants throughout all phases of anesthesia, but was never power-law distributed for patients with UWS, regardless of spatial scale. Node size distribution was power-law distributed for both. Study did not meet (Clauset et al., 2009)criteria for power law. • Criticality would not be needed for wakefulness alone but would underlie the brain’s ability to recover from anesthesia or deep sleep. Future research should investigate whether power laws or other markers of SOC are helpful predictors of recovery from coma or other minimally conscious states
Thiery et al., 2018	7 adults receiving sevoflurane anesthesia	EEG	DFA	• Unconsciousness under sevoflurane was associated with increases in LRTC in beta amplitude over frontocentral channels and decrease in alpha amplitude over occipito-parietal channels. • LRTC and oscillation amplitude may reflect different properties of the brain that are impacted during anesthesia.

*ECoG, electrocorticogram; fMRI, functional magnetic resonance imaging; EEG, electroencephalogram.*

Another area of ongoing research is the use of criticality signatures to distinguish persistent coma from other forms of unconsciousness, including deep sleep and anesthesia-induced unconsciousness. A function magnetic resonance imaging (fMRI) study compared both blood oxygen level-dependent (BOLD) signals and brain networks between patients undergoing anesthesia and patients in unresponsive wakefulness syndrome (UWS or persistent coma) (Liu et al., 2014). While the node size in these brain networks was power-law distributed in both patient groups, the node degree distribution (i.e., the number of connections between nodes in the brain network) was only power-law distributed in the healthy patients undergoing anesthesia. The authors offer a helpful analogy from the world of airports and air traffic control. In patients under anesthesia, both the airport size and the air traffic follow a power-law distribution. But in patients with UWS, the airport size remains power-law distributed while the air traffic loses its power-law distribution. This loss may reflect changes in underlying neuronal network topology that give UWS such a poor prognosis. However, more research in this area is required since the node degree distribution in this study does not meet the power-law criteria of Clauset et al. (2009) and, therefore, may follow a different distribution. In fact, another study found an exponentially truncated power law for both patients groups (anesthetized and UWS patients), which suggests the absence of a distinctive signature for the UWS group (Achard et al., 2012). Significant technical differences between Liu et al. (2014) and Achard et al. (2012) make a direct comparison difficult. Nevertheless, these studies raise the possibility that SOC underlies the brain’s ability to rebound quickly from anesthesia or deep sleep, but not from UWS or other major brain insults. Future research in this area will benefit intensivists and neurologists looking for prognostic markers of irreversible brain damage.

### Epilepsy

Epilepsy, a disorder characterized by multiple seizures and affecting 1% of the world’s population, represents a significant clinical challenge (Fiest et al., 2017). There is a robust body of work examining the applications of critical phenomena to epilepsy, as summarized in Table 2. From this literature, the following four topics emerge as clinically relevant areas of ongoing research: seizure prediction, seizure localization, seizure characterization, and quantitative analysis of seizure genesis and termination.

**TABLE 2:** Summary of epilepsy-related criticality literature.

Study	Study population	Modality	Analysis	Main findings
Worrell et al., 2002	7 adult patients with medication-resistant temporal lobe epilepsy	Interictal ECoG	Power-law estimation	• Study found evidence of SOC in interictal epileptiform discharges and suggested SOC-based method for identifying seizure focus.
Cerf et al., 2004	6 pediatric patients with presurgical epilepsy evaluation	Pre-ictal and inter-ictal ECoG, scalp EEG	Critical slowing-down of pre-ictal amplitude	• Study found evidence of criticality in synchronous fluctuations up to 1 h prior to seizure onset. • Root mean square amplitude or excess energy content were suggested as possible criticality order parameters.
Parish et al., 2004	5 adult patients with unilateral mesial temporal lobe epilepsy	Wake, sleep, pre-ictal and ictal/post-ictal ECoG	DFA	• LRTC in energy fluctuations over seconds to minutes was seen in both epileptogenic and non-epileptogenic hippocampus. • DFA exponents for non-epileptogenic regions were smaller compared to epileptogenic regions, but no difference in DFA exponents was noted between pre-ictal and baseline state.
Monto et al., 2007	5 adult patients with medication-resistant neocortical epilepsy; 2 patients received lorazepam	Inter-ictal ECoG	DFA	• LRTC was present near the seizure focus, and seen prominently in beta band (14−30 Hz). Lorazepam decreased beta-band LRTC near the focus and increased LRTC in other cortical areas. • Anti-epileptic mechanism of benzodiazepines may be related to normalization or reduction of LRTC in epileptic focus and may serve as biomarker during presurgical localization of epileptic foci.
Osorio et al., 2009	60 adult patients with mesial temporal and frontal lobe medication-resistant epilepsy	Pre-ictal and ictal ECoG	Power-law estimation	• Study finds evidence of power law distribution for seizure energy and inter-seizure interval time. Moreover, study found that seizures tend to occur in clusters, obeying an Omori-type law in which the likelihood of next seizure decreases the longer the seizure-free interval. These insights suggested a strong analogy between seizures and earthquakes, which behave in a self-organized critical way.
Osorio et al., 2010	60 adult patients with mesial temporal and frontal lobe medication-resistant epilepsy	Pre-ictal and ictal ECoG	Power-law estimation	• Five statistics from seismology (including energy, inter-event waiting time, direct and inverse Omori law, time to next earthquake) were compared to analogous statistics in seizures. Insights from SOC in earthquakes were applied to seizures.
Hobbs et al., 2010	6 pediatric epilepsy patients	LFPs from removed epileptic brain tissue	Branching parameter, correlation, power-law estimation	• Some epileptic brain tissue exhibited prolonged hyperactivity. Study found positive correlation between firing rate and critical branching parameter during this prolonged hyperactivity, suggesting possible existence of positive feedback loop in some forms of epilepsy.
Meisel et al., 2012	8 adult patients with focal epilepsy	Pre-ictal, ictal, post-ictal ECoG	Power-law estimation*	• Study found a robust power law distribution of phase-locking intervals and saw this as evidence of SOC. Variations in goodness-of-fit suggested that not all brain regions are tuned to criticality at the same time. • Significant deviation from power law during seizure suggested departure from critical state, in part due to excessive synchronization.
Meisel and Kuehn, 2012	8 adult patients with intractable epilepsy	Pre-ictal, ictal ECoG	Variance of signal amplitude	• The inverse of the signal variance followed a scaling law and decreased as a seizure approached. • Oscillations in variance leading toward seizure onset were suggestive of critical transition characterized by a Hopf bifurcation.
Kramer et al., 2012	19 adult patients with different epilepsy etiologies	ictal and post-ictal from scalp EEG, ECoG, LFP and MUA	Critical slowing down, temporal and spatial correlations, flickering	• Multi-scale analysis suggested seizure termination happens through a critical transition, modeled by a discontinuous fold bifurcation. • Status epilepticus may represent a system’s inability to cross a critical transition, instead reverberating between ictal and post-ictal attractors.
Roberts et al., 2014a	13 term neonates with either birth hypoxia or circulatory collapse	Non-ictal EEG of burst suppression seen within 18 h of birth	Power-law estimation*, scaling relations, burst shape analysis (skewness, kurtosis)	• Power law relationship was seen between burst size and duration. The scaling exponent of that relationship increased as burst suppression gave way to normal EEG activity. • Shape analysis revealed leftward skewness, also seen in crackling noise like Barkhausen noise. Skewness resolved as burst suppression gave way to normal EEG activity. Leftward skewness may be related to state-dependent metabolite depletion. • Other signs of criticality include evidence of shape scaling function and inter-relationship of power-law exponents for burst area, duration, and area-duration relationship. There is evidence of critical phase transition from burst suppression to resumption of normal EEG.
Cook et al., 2014	15 adult patients with refractory epilepsy	ECoG recorded over 0.5−1.8 years	Power-law estimation; Hurst exponent	• Study found evidence of a power law for inter-seizure interval in large human dataset, with scaling exponent −1.5 consistent with previous studies. Hurst analysis was consistent with a long-memory process in most subjects, with memory ranging from 3 to 40 days. • The presence of long-memory implies a less complex and more predictable system−epilepsy prediction may depend on the existence (or not) of long-memory in different types of epilepsy.
Minadakis et al., 2014	2 adult patients (iEEG); 6 adult patients (scalp EEG)	Pre-ictal and ictal ECoG and scalp EEG	Q-parameter, Tsallis entropy, volumetric energy density	• While seizures showed consistently elevated q-parameter in range 1.6−1.8, inter-ictal and pre-ictal EEG could not be readily distinguished by q-parameter alone. • There was evidence of intermittent criticality (IC), which may generalize to SOC on larger time scales. Tsallis entropy did not change significantly from pre-ictal to ictal, suggesting other forms of complexity may be involved in the ictal period. The concept of “fractures and faults in the brain,” a continuation of the earthquake-seizure analogy, may be a fruitful framework for advancing seizure prediction.
Yan et al., 2016	3 adult patients with refractory temporal lobe epilepsy	Pre-ictal, ictal, post-ictal ECoG	Power-law of wavelet spectral density, Hurst exponent, linear correlation coefficient	• The pre-ictal to ictal transition was characterized by transition from anti-correlation to correlation as given by Hurst exponent and fractional Brownian motion (fBm) model. • Hurst exponent changes in ictogenesis happened throughout the brain and not just at epileptic foci. In posti-ical state, high Hurst exponents were seen throughout the brain, suggesting seizure was result of breakdown of global neuronal network. • Wavelet-based spectral density approach in setting of fBm model may be helpful tool for seizure prediction.
Arviv et al., 2016	12 adult and 8 pediatric patients with refractory epilepsy; 18 age-matched healthy controls	Inter-ictal MEG	Power-law estimation*, branching parameter, avalanche shape analysis	• Patients with drug-resistant epilepsy showed deviations from expected branching parameter at criticality, especially at interictal epileptiform discharges. • Quantitative analysis of MEG using criticality-related parameters may allow better evaluation of excitation-inhibition balance in sleep-related disorders and in epilepsy.
Witton et al., 2019	2 pediatric and 1 adult patients with medication-resistant epilepsy	MEG, beamformer source models, volumetric maps	Hurst exponent, rogue wave analysis, kurtosis of inter-ictal spikes	• Hurst exponent analysis, kurtosis, and rogue waves could serve as important parameters in automatic classifiers for epilepsy detection, as well as for patients referred for pre-surgical MEG evaluation who do not have interictal spikes. • Epileptiform activity was strongly persistent, suggesting that Grainger causality is not suitable for epilepsy data.

Asterisk (*) represents power-law estimations that meet criteria equivalent to or more stringent than Clauset et al. (2009). MEG, magneto-encephalography; ECoG, electrocorticogram; LFP, local field potential; MUA, multi-unit activity; DFA, detrended fluctuation analysis.

Seizure prediction is an important goal in epileptology (Mormann et al., 2007). Efficient seizure prediction, even if only by a few seconds to minutes, may allow patients enough time to administer anti-epileptic medications before seizure onset, either by themselves or through automated implanted devices (Cook et al., 2013). To this end, several studies have identified criticality-based signatures that may help predict the onset of a seizure. For example, critical systems near a phase transition will exhibit signs of “critical slowing.” A combined EEG and ECoG study of children with epilepsy found evidence of such critical slowing in synchronous fluctuations up to 1 h before seizure onset (Cerf et al., 2004). Other studies have taken advantage of an uncanny similarity between seizures and earthquakes. Seizures, like earthquakes, exhibit several properties that are characteristic of SOC (Bak and Tang, 1989; Bak et al., 2002). For example, both seizures and earthquakes cluster temporally, such that the likelihood of the next seizure or earthquake decreases the longer the seizure-free or earthquake-free interval (Omori, 1895; Sornette and Sornette, 1989; Osorio et al., 2009, 2010). Studies have identified other SOC properties in seizures. These include a power-law distribution of inter-seizure intervals (Osorio et al., 2009, 2010; Cook et al., 2014) and of time intervals between non-ictal epileptiform discharges, including burst suppression in neonatal hypoxia (Worrell et al., 2002; Roberts et al., 2014a). By better characterizing these properties, one could hope to identify parameters that can help predict a future seizure−much the same way that seismologists would like to predict the next earthquake (Meisel and Loddenkemper, 2019).

The predictability of these seizures may be related to increased temporal correlation or long memory. A study of long-term ECoG recordings found that epileptiform discharges and seizures in some patients were consistent with long-memory processes, with signal correlations going as far back as 40 days before seizure onset (Cook et al., 2014). Long memory suggests a decrease in signal complexity and, thus, more predictability. These studies open up the possibility of using LRTC and other correlation measures to predict impending seizures. While there is no gold-standard approach for seizure prediction at this time, the brain criticality framework offers new insights that will hopefully produce several candidates for effective seizure prediction. Some of these candidates include excess energy content of EEG signals (Cerf et al., 2004), heavy-tailed distributions of inter-ictal discharges (Osorio et al., 2010), signal variance (Meisel and Kuehn, 2012), Hurst exponent analysis (Cook et al., 2014; Yan et al., 2016), q-parameter, and volumetric energy density from Tsallis non-extensive statistical mechanics (Minadakis et al., 2014).

The surgical removal of epileptic foci for treating refractory epilepsy requires adequate localization of the source of epileptiform discharges (Mu et al., 2014). Localizing the epileptic focus remains a challenging part of this process. Epileptic foci exhibit many critical features, like power-law distributions and LRTC, that are useful for localization. In an ECoG study of patients with temporal lobe epilepsy, the epileptic foci produced a power-law-like behavior of seizure energy and inter-seizure intervals (Worrell et al., 2002). An ECoG study of epileptic patients found that both non-epileptic and epileptic foci in the hippocampus exhibited LRTC (Parish et al., 2004). However, the epileptic foci had larger scaling exponents compared to the non-epileptic foci. Another ECoG study found a similar result, namely stronger LRTC near the seizure focus (Monto et al., 2007). More recently, a magnetoencephalography (MEG) study found that the Hurst exponent – a metric of LRTC – improved the detection of seizure foci (Witton et al., 2019). These studies globally suggest that observables of critical phenomena, like power-law regimes and increased correlation, can improve seizure localization techniques.

The lens of criticality is also casting new light on the characteristics and dynamics of seizures themselves. Several publications have argued that seizures and inter-ictal epileptiform discharges, including burst suppression, represent a critical phenomenon that is power-law distributed (Worrell et al., 2002; Cerf et al., 2004; Osorio et al., 2009, 2010; Roberts et al., 2014a). If this is correct and the reported power-law exponents for seizure energy are between 2 and 3 (as many are), then it follows that, at least mathematically, seizures ought to have a finite mean (energy, size, duration) but infinite (energy, size, duration) variance. Some have argued that this may account for status epilepticus, the phenomenon of prolonged seizures lasting hours to days. The theoretically infinite variance of seizure energy would lead to prolonged seizures that would ultimately resolve because of the finite metabolic supply available to neurons (Osorio et al., 2010; Roberts et al., 2014b). Moreover, if seizures represent a power-law distributed critical phenomenon, they cannot be described by their mean values, since, in a scale-free distribution, there is no “typical” value or mean. Thus, there may be no point in reporting mean seizure duration or energy in clinical publications. Rather, in future clinical and epidemiological studies, it may be more pertinent to report power-law exponents, which best characterize this distribution (Osorio et al., 2009).

However, other publications have argued that the normal brain at rest is in a critical state. Therefore, seizures and interictal epileptiform activity should represent a departure from criticality. This departure from criticality perhaps arises from synchronization effects and characteristic scales present in seizures that become dominant, thus diminishing the scale-free distribution (Osorio et al., 2009; Meisel et al., 2012; Arviv et al., 2016). This departure from a scale-free distribution has been confirmed visually with a “knee,” “shoulder,” or “bump” – different words for the same anomalous deviation−in the log-log plot of several probability density functions, including that of seizure energy (Osorio et al., 2009), phase-locking intervals (Meisel et al., 2012), neuronal avalanche size (Arviv et al., 2016) and burst area in burst suppression (Roberts et al., 2014a). The evidence suggesting that the resting brain is in a critical state is strong (Kitzbichler et al., 2009; He, 2011; Tagliazucchi et al., 2012; Daffertshofer et al., 2018), even if some of that evidence (Kitzbichler et al., 2009; Meisel et al., 2012) has been challenged (Botcharova et al., 2012). Seizures would logically seem to represent a departure from the resting state of the brain and thus from a critical state. How then can one reconcile this with all the evidence suggesting that seizures behave like a critical phenomenon?

Perhaps one way to reconcile this information is to look more closely at the types of variables (Milton, 2012). In cases that have identified power-laws in the resting brain (with departures during seizures), the variables studied were usually neuronal avalanche size and duration, in what could be called an “avalanche approach.” On the other hand, studies that found power-law behaviors of seizures took more of an “earthquake approach” in which the variables were usually seizure energy and inter-seizure interval (Worrell et al., 2002; Osorio et al., 2009). Since different properties, or “laminar phases,” are being examined in each case, it may not be reasonable to compare their power-law exponents (Milton, 2012).

Moreover, the range of power-law exponents found in both the avalanche and earthquake approaches is broad and overlapping. The exponents encompass the range of –3/2, which is expected for avalanche size in SOC, up to −5/3, characteristic in turbulent dynamical systems (Milton, 2012). This broad range may result from particular experimental conditions (including digitization rates of instruments). But this range may also reflect the reality that neurons, unlike earthquakes and sand-piles, learn and adapt (Bonachela et al., 2010). The existence of characteristic scales (“bumps”) on log-log plots, which perturb the expected scale-free distribution, may also be due to characteristic scales from rare neurological events such as dragon-kings (Pisarenko and Sornette, 2012; Sachs et al., 2012). Finally, since many control parameters may be involved in governing these systems, the possibility of “double criticality” whereby critical regimes coexist with different order and control parameters may also be at work in this apparent disagreement (Hesse and Gross, 2014). Resolving this disagreement on both theoretical and experimental grounds will be an important area of future research.

Brain criticality also offers insights into seizure initiation. Epileptic foci removed from pediatric epilepsy patients exhibited neuronal hyperactivity, whose increased firing rate correlated with an increased branching parameter (Hobbs et al., 2010). This finding suggests that in some epileptic syndromes, a positive feedback loop between firing rate and branching parameter may be responsible for generating seizures as a super-critical state. An ECoG study found oscillations in signal variance in the lead-up to a seizure (Meisel and Kuehn, 2012). These pre-ictal oscillations were suggestive of a critical transition, characterized mathematically by a Hopf bifurcation. Despite the small number of studies in this area, research on seizure generation using criticality is promising.

Several studies suggest that seizure termination may also involve a critical transition. In one study by Kramer et al. (2012) the brain’s inability to complete a critical transition results in status epilepticus, in which the brain dynamics constantly reverberate between the ictal and post-ictal state (i.e., attractor), without ever crossing the threshold that effectively ends a seizure. In a study of neonates with birth hypoxia, researchers found evidence of a critical phase transition in the shift from burst suppression to the resumption of normal EEG patterns (Roberts et al., 2014a). The role of benzodiazepines in seizure termination may also be related to criticality. In a small ECoG study of patients with epilepsy, study authors found that a decrease in LRTC in the ictal focus accompanied the clinical resolution of a seizure after benzodiazepine administration (Monto et al., 2007). These studies all suggest that criticality plays a role in seizure termination.

### Neurodegeneration

A small number of studies (see Table 3) have examined the role of critical phenomena in neurodegenerative diseases, like Alzheimer’s disease (AD) and Parkinson’s disease (PD). These studies reveal new insights about the pathophysiology of these diseases and suggest novel markers for disease monitoring.

**TABLE 3:** Summary of neurodegeneration-related criticality literature.

Study	Study Population	Modality	Analysis	Main Findings
Stam et al., 2005	24 adults with AD, 19 non-demented adults with subjective memory complaints	EEG during eyes-closed resting state	DFA	• Study examined mean synchronization in different frequency bands. Mean EEG synchronization and spontaneous fluctuations of synchronization were lower in AD in upper alpha and beta bands compared to non-AD patients. Mean synchronization level and DFA exponents were correlated to MMSE score. Both patients and controls showed scale-free patterns of synchronization fluctuations, extending to up to 10 s. • AD patients may have brain electrical pattern consistent with SOC but exhibit decreased processing speed from decreased fluctuations of synchronization.
Montez et al., 2009	19 adults with early-stage AD, 16 age-matched controls	MEG during eyes-closed resting state	DFA, burst statistics	• Using criticality-based “avalanche analysis,” study found that AD patients had a strongly reduced incidence of alpha-band oscillation bursts over temporo-parietal regions and markedly weaker autocorrelations on long time scales (1–25 s). • Study suggested that criticality-related measurements of amplitude dynamics of oscillations may prove useful as neuroimaging biomarkers of early-stage AD.
Hohlefeld et al., 2012	10 adults with idiopathic PD	LFP from bilaterally implanted electrodes from STN DBS	DFA	• Study examined LRTC of the amplitude envelope of LFPs recorded from subthalamic nucleus, both on and off of levodopa. “On levodopa” state was characterized by stronger LRTC (up to 50 s) than the “off” state in beta and high-frequency oscillations. • Weaker LRTC in off state might indicate limited information processing in dopamine-depleted basal ganglia. Study suggests LRTC may serve as possible biomarker of pathological neuronal processes in PD.
Ruiz et al., 2014	1 adult with severe idiopathic PD, treated with STN DBS	Inter-onset interval (time between note onset of two subsequent notes) while playing piano, with STN DBS both on and off	DFA, spectral density	• Study investigated temporal deviations during skilled piano performance of a non-professional pianist with severe PD treated with STN DBS. In tremor-affected right hand, timing fluctuations of the performance exhibited random correlations while off DBS. When DBS was on, LRTC increased along with general motor improvement. • The authors remark that the presence of LRTC and *1/f* laws in performance (improved by DBS) can be related to the brain operating near criticality.
Vyšata et al., 2014	110 adults with moderate-to-severe AD, 110 healthy controls	EEG during resting-state	Power-law estimation, spectral density	• Study evaluated power-law exponents for power-law distribution of EEG spectrogram from patients with AD compared to healthy controls. Power-law exponent was found to be a specific marker of AD in the frontal EEG channels. Authors suggest that loss of functional connectivity may explain these differences in power-law exponents. Clinical utility of power-law exponent of spectrogram would require repeating the study on patients with mild cognitive impairment or early stages of AD.
West et al., 2016	12 adults with PD who received bilateral STN DBS	LFP while on and off dopaminergic medications	Spectral density, signal coherence, DFA	• Study examined LFPs from PD patients undergoing STN DBS surgery, on and off of dopaminergic medications. Authors demonstrated up-modulation of alpha-theta (5−12 Hz) band power with L-DOPA treatment, whilst low beta band power (15−20 Hz) band-power was suppressed. Using DFA adapted to phase synchrony (DFA-PS), study found LRTC in phase dynamics of coupled left and right STN region for low beta band. Low beta band DFA-PS scaling exponent magnitude for interhemispheric pairs was positively correlated with PD symptom severity in the off-medication state. Findings suggested that the more severe the motor impairment, the closer the subthalamic network was to onset of synchronization, implying shift of network toward supercritical regime.

*AD, Alzheimer’s disease; PD, Parkinson’s disease; STN DBS, subthalamic nucleus deep brain stimulation; MMSE, Mini-Mental Status Examination; LFP, local field potential.*

Cognition requires production and subsequent decay of synchronization in neural networks (Breakspear and Terry, 2002). Moreover, in healthy adults, spontaneous fluctuations in synchronization are known to follow a power-law distribution, suggestive of an underlying SOC state (Stam and de Bruin, 2004). Does AD represent a departure from critical dynamics? Is synchronization perhaps a control parameter given its importance in cognition? To begin to answer these questions, researchers compared various measures of synchronization on EEG between patients with AD and non-demented patients with subjective memory problems (Stam et al., 2005). While both cohorts maintained a scale-free distribution of spontaneous fluctuations of synchronization, the mean synchronization and its fluctuations were both decreased in the upper alpha and beta frequency range in the AD patients compared to the non-demented patients. This finding is consistent with the view that AD patients maintain a SOC state but with decreased ability to generate and destroy synchronized neural networks. The authors go on to speculate that perhaps synchronization loss in the upper alpha and beta band is one of the first quantifiable changes in AD since it is statistically different from non-demented patients who report memory impairment. Moreover, the mean synchronization level and the DFA exponent of synchronization fluctuations were both correlated to the Mini-Mental State Examination (MMSE) score. Synchronization-based metrics may, therefore, prove helpful for diagnosis and monitoring of early-onset AD.

Other metrics inspired by brain criticality show promise for the diagnostic evaluation of early-onset AD. A MEG study discovered a decreased incidence of alpha-frequency oscillation bursts and weaker auto-correlations in patients with early-onset AD compared to controls (Montez et al., 2009). Its authors concluded that oscillation amplitude dynamics may be beneficial for early-onset AD detection. Operating from the framework of SOC, a large, resting-state EEG study of patients with moderate-to-severe AD compared to healthy controls also identified a possible marker for early AD detection (Vyšata et al., 2014). The power-law exponents for spectral densities (per brain region) were compared between AD and healthy patients. A statistically significant difference in the power-law exponent in the frontal and pre-frontal lobes was noted. This result is perhaps not so surprising given that frontal lobe atrophy typically accompanies AD dementia. Interestingly, the most highlyspecific and predictive area of the brain for AD in this study was the temporal region (Vyšata et al., 2014). Future studies using this approach and focusing on the temporal region may be able to validate this power-law approach as a diagnostic metric in patients with early manifestations of AD. To this point, a recent fMRI-connectome study showed that a combination of criticality-based metrics can help distinguish neurotypical adults from those with mild cognitive impairment or AD (Jiang et al., 2018).

Parkinson’s disease (PD) may be a case of how deviation from a critical state in crucial motor circuits leads to motor symptoms like tremors, bradykinesia, and rigidity. Researchers have identified LRTC from the subthalamic nuclei (STN) of patients with PD undergoing deep brain stimulation (DBS) (Hohlefeld et al., 2012). These correlations increased with the administration of levodopa, one of the common medications for treating PD. In a rat model of PD, LRTC also increased following administration of apomorphine (Cruz et al., 2009), which suggests that restoration of LRTC may be related to symptomatic improvement in PD. A study of PD patients who underwent DBS surgery found LRTC in the dynamics of the bilateral STN, both on and off medications (West et al., 2016). Using an adaptation of DFA to study synchronization called DFA-PS (Botcharova et al., 2015), the authors found that the DFA-PS exponent was positively correlated with motor symptom severity when patients were not receiving dopaminergic medications. Therefore, these authors suggested that patients with more severe motor symptoms are closer to the onset of pathological synchronization, which may reduce effective information transfer in these important neural circuits (Hanslmayr et al., 2012; West et al., 2016). In this regard, PD may represent a situation of departure from a critical state toward perhaps a hyper-synchronized supercritical state.

Obtaining recordings from deep brain structures like the basal ganglia is not usually possible outside of DBS. Since DBS is not the first-line therapy for PD, it seems unlikely that LRTC will develop into a helpful marker of PD onset and progression. Gait analysis and related behavioral metrics, on the other hand, may offer a more convenient way to follow the clinical evolution of PD. Healthy human gait is characterized by *1/f* noise, which is known to be a feature of SOC systems (Hausdorff, 2009). This *1/f* noise disappears in PD but resumes with non-invasive auditory rhythmic stimulation (Hove et al., 2012). In a unique study, a single patient with idiopathic PD and right-handed tremor, who happened to be an accomplished pianist, received DBS and was subsequently asked to perform works of piano both with and without active DBS (Ruiz et al., 2014). Without DBS, correlations in the inter-onset interval (i.e., the time between note onset of subsequent piano notes) were random. But with active DBS, long-range correlations of inter-onset interval emerged, along with general motor improvement of the affected right hand on the Unified Parkinson’s Disease Rating Scale (UPDRS) - III scale. The authors suspected that DBS provides a similar stimulus to the non-invasive rhythmic stimulation of Hove et al.’s (2012) experiment, which restores the *1/f* noise of gait, presumably by restoring a critical state in the motor basal ganglia circuit (Ruiz et al., 2014). Future research should focus on establishing whether restoration of *1/f* noise is necessary for motor improvement in PD and whether gait-based metrics can serve a clinical purpose in either diagnosing or monitoring PD.

### Neurodevelopment

The brain criticality hypothesis also applies to the newborn brain that matures and ages across an average human lifespan. As it grows, the brain’s electrical signature undergoes changes that are helpful markers of typical development. Research in this area has taken shape around two central themes (see Table 4). The first is the study of brain oscillations in premature infants or infants with birth asphyxia (hypoxemic-ischemic encephalopathy or HIE). The other is the study of brain oscillations in children, adolescents, and adults to characterize the electrical patterns that correlate with structural and anatomic changes of aging.

**TABLE 4:** Summary of neurodevelopment-related criticality literature.

Study	Study population	Modality	Analysis	Main findings
Suckling et al., 2008	22 healthy adults (11 in the age range 20−25 years old, 11 in the age range 60−70 years old), matched for education.	Task and resting-state fMRI; double-blind, randomized administration of subcutaneous scopolamine or saline (placebo)	Hurst exponent, singularity spectrum using wavelet transform maximum modulus method, multifractal parameters	• Previous research had shown that healthy aging and cholinergic blockade with scopolamine were associated with increase in Hurst exponent, implying a marker of suboptimal neurophysiological dynamics (Wink et al., 2006). However, previous research had also shown that faster processing speed in certain tasks also led to increased Hurst exponent (Wink et al., 2008). This study used multifractal approach to tease apart the discrepancy and used (Castaing et al., 1990) algorithm to identify the role of turbulence. Authors conclude that turbulence has limited validity, while invariance of energy dissipation is better explained by critical phenomena.
Thatcher et al., 2009	458 healthy pediatric subjects (age 2 months to 16 years old)	resting-state EEG	Mean phase shift duration, phase-locking intervals, power-law estimation, spectral density analysis	• Study explored development of SOC as measured by EEG phase reset−a combination of phase shift followed by phase stability (or phase locking) – from infancy to adolescence. Mean duration of phase locking (150−450 s) and phase shift (45−67 s) increased as a function of age. Development and number of synaptic connections may be a possible order parameter for SOC during human brain maturation.
Berthouze et al., 2010	36 healthy subjects (age 0–55 years old)	EEG during wrist-extension task	Spectral density analysis, DFA	• In physical systems, SOC states take time to develop. Study found that there is a scale-free nature to EEG LRTCs from early childhood through to maturity but that the magnitude of these effects changed with age.
Smit et al., 2011	1433 healthy subjects (age 5–71 years old)	Resting-state EEG	DFA, spectral density, principal component analysis	• Study observed significant increases in LRTC from childhood to adolescence and into early adulthood. PCA of the spatial distribution of LRTC showed functional-anatomic segregation between frontal, occipito-temporal, and central regions that became more integrated with development. DFA scaling analysis may be useful as a biomarker of pathophysiology in neurodevelopmental disorders like ADHD and schizophrenia.
Hartley et al., 2012	11 pre-term newborns (23−30 weeks gestation)	EEG	Hurst exponent (Whittle estimator and DFA)	• LRTC were identified in very pre-term infants through two estimate of Hurst exponents of EEG bursts. The study found no difference in Hurst exponents between subjects with and without brain hemorrhages, indicating that despite lower burst event frequency for newborns with hemorrhages, signal complexity was maintained. Overall EEG pattern was suggestive of relaxation dynamics as can be seen near a phase transition.
Mares et al., 2013	17,722 healthy adults (ages 18−70 years old)	Resting-state EEG	Spectral density, power-law estimation	• Study investigated parameters of colored noise in EEG in healthy adults. Absolute value of power spectra exponent decreased significantly with age, perhaps indicative of age-related changes in self-organization of brain activity due to brain atrophy. Globally, there was a trend from pink noise to white noise with age that was seen consistently in beta and delta bands.
Fransson et al., 2013	fMRI: 18 term newborns and 17 healthy adults (ages 22−41 years old); EEG: 15 term or post-term newborns, 7 healthy adults (ages 14−53 years old)	EEG in stage 2 sleep, fMRI	power-law estimation	• Study found that newborn brain dynamics follow apparently scale-free frequency power distribution across several orders of magnitude in both fMRI and EEG signals. In newborns, primary sensory areas exhibit larger power-law exponents than higher associative cortical areas, in contrast with the adult brain. • High power-law exponents in newborns were likely due to spontaneous activity transients (SATs) or bursts that seem to underlie brain activity in the first neuronal networks in the human brain (Vanhatalo and Kaila, 2006).
Thatcher et al., 2014	70 healthy subjects (age 13−20 years old)	LORETA (EEG) of the Brodmann areas of the default mode network in the delta frequency band	Phase shift duration, phase lock duration	• Study found no significant correlation between age and phase shift and phase lock duration from EEG of the default mode network. Study findings were globally consistent with SOC.
Frohlich et al., 2015	39 preschool-age healthy subjects	EEG	Frequency variance, power-law estimation	• Study quantified variance of rate of change of signal phase (i.e. frequency variance) as a proxy for phase reset (or signal stability). Frequency variance increased with age in preschool age children. This method is helpful in pediatric studies because it does not require long recordings. Authors suggest that phase resets are critical fluctuations driven by SOC.
Iyer et al., 2015	43 preterm neonates (23−28 weeks gestation)	Resting-state single-channel EEG recorded at 12, 24, 48, and 72 h of life	Power-law estimation*, burst shape analysis, generalized linear model	• Study found scale-free properties of EEG bursts in extremely preterm infants as soon as 12 h after birth. Metrics of burst shape were predictive of neurodevelopmental outcomes using Bayley scales. Specifically, symmetric bursts that are relatively flat at long time scales suggested a favorable neurodevelopmental outcome. Conversely, skewed and highlykurtotic bursts in neonates shortly after birth were suggestive of long-term
				disability. Low burst slope values, moderated by effect of gestational age, correlated with poor scores on the Bayley scales or early death.
Padilla et al., 2020	33 children born extremely prematurely and 29 children born term	fMRI and diffuse MRI at 10 years old	Ignition analysis, structural and connectivity matrices, whole-brain Hopf model	• Study compared 10 year-old children who were either born extremely pre-term (EPT) or born at term, using fMRI with ignition analysis. Intrinsic ignition events allow propagation of neuronal activity to other regions over time which drives global integration. Extremely pre-term children had reduced intrinsic ignition events, consistent with previous study that had shown reduced spontaneous neuromagnetic activity in pre-term children. Study found that the hierarchy of information processing based on the variability of intrinsic ignition events was predominantly driven by visual and sensory region in EPT children compared to the higher-order processing areas like the fronto-temporal region and the associative area in term children.
Jannesari et al., 2020	19 term infants	High-density EEG during an oddball auditory task	Power-law estimation*, DFA	• Study evaluated infants at 6 and 12 months of age during auditory odd-ball task to see if the bursting, scale-free activity of pre-term infants continues as scale-free avalanche activity outside the newborn period. Suprathreshold events organized as spatiotemporal clusters whose size and duration were power-law distributed while time series of these events showed significant LRTCs. Power law was a better distribution fit than log-normal and exponential. No significant differences were noted between 6 and 12 months, suggesting stability of avalanche dynamics and LRTCs in the first year after birth.

Asterisk (*) represents power-law estimations that meet criteria equivalent to or more stringent than Clauset et al. (2009). LORETA, low-resolution electromagnetic tomography; PCA, principal component analysis; ADHD, attention deficit hypersensitivity disorder; MDI, motor development index.

If the mature human brain exhibits signs of criticality, it seems reasonable to ask whether those signs are also present in the term or pre-term neonate. Studying burst activity on the EEG of pre-term infants, Hartley et al. identified LRTC and dynamics that were suggestive of a phase transition (Hartley et al., 2012). Despite several infants having intracranial hemorrhages, the Hurst exponents describing the LRTC were similar for infants with and without bleeding. This finding suggested that the brain maintains temporal complexity despite this vascular insult. Another EEG study showed that power-law exponents of electrical bursts were predictive of neurodevelopmental sequelae in term infants with HIE (Roberts et al., 2014a). This fascinating discovery led to a similar EEG study in extremely preterm infants, searching for criticality-based metrics that could predict long-term sequelae. A careful analysis of several parameters from shape analysis led to the identification of the slope of burst shape, among other parameters, that could serve as a sensitive and specific predictor of neurocognitive and motor sequelae in this unique population (Wikstro et al., 2015). In sum, shape analysis and power-law exponents predict clinical outcomes in preterm and term infants, respectively. Regardless of hemorrhage status, the brains of preterm infants exhibit LRTC. These findings suggest that criticality plays a vital role in the dynamics of the preterm and term infant brain.

How criticality or near-criticality emerges and evolves in the infant’s brain through adolescence into adulthood is another crucial area of investigation. Jannesari et al. (2020) studied term infants at 6 and 12 months of life using high-density EEG to see if scale-free brain activity that was already known to exist at birth (Fransson et al., 2013) continued in the first year of life (Jannesari et al., 2020). At both 6 and 12 months, the EEG organized spatiotemporally according to a power-law with significant LRTC. The authors found no significant differences between the 6- and 12-month data, suggesting a degree of stability in neuronal avalanche dynamics after the newborn period. But while there do not seem to be significant differences in LRTC and power-law distributions during the first year of life in term babies, a significant difference does exist between pre-term babies and term babies, even a decade after birth. Using functional and diffusion MRI, researchers examined the brain dynamics of 10-year-old children, roughly half of whom were born extremely premature, and the other half at term (Padilla et al., 2020). Extremely premature (EP) children generated fewer electrical events (i.e., decreased ignition) compared to the term children – suggesting decreased global integration from decreased firing to other important brain regions over time (Deco et al., 2017). EP children also exhibited abnormal hierarchical organization, which autistic children are also known to exhibit (Parr et al., 2018), further linking prematurity and autism (Padilla et al., 2017). EP children’s brain dynamics showed decreased synchrony and sub-criticality compared to term children, mainly in brain areas with rich-club architecture (Ball et al., 2014). This difference in synchronization perhaps occurs due to abnormal development of white matter in EP children (Uhlhaas et al., 2010). Yet, whereas extreme prematurity seems to predispose to some degree of sub-critical brain dynamics, it seems from other studies that term infants go on to exhibit similar power-law-like brain dynamics as adults. In an EEG-fMRI study, newborn brain dynamics followed scale-free power (frequency) distributions across several orders of magnitude, with larger power-law exponents in primary sensory areas compared to associative cortical areas (Fransson et al., 2013). In contrast, power-law exponents for adult brain dynamics were highest in associative cortices. While both adults and term infants exhibit scale-free dynamics, the increased exponents in different brain areas suggest that development and aging control, to some degree, the cartography of near-criticality in the brain.

As for aging, the development of criticality and its suspected decay with age have been the subjects of several studies (see Table 4). Phase reset (PR), a combination of phase-shift followed by phase-locking of EEG signals, is a powerful marker of SOC used in many age-based studies of criticality. In a study of PR in close to 460 subjects in the age range of 2 months to 16 years old, PR followed a *1/f* distribution, with a longer mean duration of both phase-shift and phase-locking as a function of age (Thatcher et al., 2009). The presence of this distribution argues in favor of scale-invariant fluctuations in PR, consistent with SOC (Thatcher et al., 2009, 2014). In a study of pre-school children, EEG frequency variance, a proxy for PR, was also power-law distributed and increased with age (Frohlich et al., 2015). Like PR, frequency variance may reflect critical fluctuations driven by SOC.

LRTC has also been found to correlate with aging. A study of about 1430 subjects from ages 5 to 71 revealed significant increases in LRTC from childhood through adolescence into early adulthood (about age 25), after which LRTC stabilized (Smit et al., 2011). Scale-free modulations of resting-state oscillations, therefore, seem to reflect brain maturation. Moreover, principal component analysis (PCA) showed progressive integration of segregated functional-anatomic brain regions with age – consistent with increased spatial correlation from critical dynamics. In a smaller study of subjects ages 0 to 55, EEG LRTC was present from early childhood into adulthood but with magnitudes that changed differently depending on age, EEG electrode, and frequency band (Berthouze et al., 2010). For example, LRTC magnitude increased with age in the beta band in central and parietal electrodes but decreased with age in the theta frequency range. But temporal correlation is not the only observable that appears to decrease with age in some frequency bands. A study of resting-state EEG in nearly 18,000 individuals ages 18 to 70 found a decrease in the power-law exponent of spectral density with age (Vyšata et al., 2014). The authors theorized that brain atrophy with age might lead to loss of neuronal connections (Morrison and Hof, 1997), which would shift the neural networks away from their scale-free topology (Barabási and Albert, 1999) and thus away from power-law dynamics.

While there may be some appearance of contradiction in these results (i.e., increased LRTC in one frequency range versus another with aging), there is precedent for contradictory results finding resolution when the right tool is applied. For example, both healthy aging and anticholinergic medications increase the Hurst exponent (Wink et al., 2006). Yet, faster processing speeds, which are not characteristic of aging, also lead to increases in the Hurst exponent (Wink et al., 2008). Suckling et al. (2008) reconciled these results with the introduction of a multi-fractal approach and demonstrated that criticality offered a better explanation than turbulence for these brain dynamics. This example highlights how the right methodology and tools can help make sense of disparate results. Methodological developments, like the index of functional criticality (Jiang et al., 2019), will hopefully generate more useful clinical results in this area. The applications of criticality to aging and geriatric medicine are still in their infancy but deserve more emphasis in a society with an increasingly elderly population.

### Cognition, Attention, Learning, and Autism

In computational and theoretical models, criticality optimizes certain features of learning, including optimal information capacity and transmission (Shew et al., 2011; Shew and Plenz, 2013; Del Papa et al., 2017). Important aspects of human learning, including cognition and attention, have been analyzed through the lens of criticality (see Table 5). Studies on attention-deficit hypersensitivity disorder (ADHD) and autism, in which features of neurotypical cognition and attention are disrupted, are few in number but appear promising.

**TABLE 5:** Summary of cognition-related criticality literature.

Study	Study population	Modality	Analysis	Main findings
Lai et al., 2010	30 male adult subjects with autism or Asperger’s syndrome, 33 age- and IQ-matched male adult controls	fMRI during resting-state	Hurst exponent	• Study examined complexity of endogenous, low-frequency neurophysiological processes in patients with ASD compared to control patients. Study confirmed that spontaneous BOLD signal fluctuations in the brain, specifically in regions implicated as atypical in previous autism neuroimaging studies, had small but significant decrease in Hurst exponents in the autistic compared with neurotypical group. This finding indicated a shift-to-randomness of brain oscillations in the autistic brain. • Though the meaning of the Hurst exponent ins limited by our understanding of neuronal and blood-supply sources to the measured BOLD signal, nevertheless fractal scaling may serve as indicator of organizational properties of local neural circuits.
Altamura et al., 2012	12 healthy adults	Response times to working memory and response tasks	Probability density functions, power-law estimation	• Power law-like behavior was noted in the upper tails of the CDF of response times for working memory tasks. This finding possibly reflects emergence of scale-free behavior in time series as an adaptation to increased cognitive requirements. Increasing cognitive load could shift random behavior to scale-free behavior near a critical point.
Dimitriadis et al., 2013	23 children with reading difficulties, 27 age- and IQ-matched children	MEG during resting-state	DFA	• Study examined MEG in children with reading difficulties compared to children without reading difficulties. Children with reading difficulties had decreased overall network organization across all frequency bands (global efficiency decrease) and a decrease in temporal correlations between sensors covering the left temporoparietal region. Study suggested that the specific parameters of SOC vary systematically in presence of reading difficulties. Both groups exhibited scale-free global network connectivity dynamics.
Tinker and Velazquez, 2014	15 children with high-functioning autism, 16 neurotypical children (ages 7−16 years old)	MEG during two executive function tasks	power-law estimation	• Study examined scaling of phase synchrony in MEG in patients with ASD compared to controls. Power-law scaling of phase synchrony was not common in either group. Its frequency of occurrence diminished with increased cognitive load/effort as children performed more difficult tasks. Power law distribution coexisted with other distributions (e.g., exponential) suggesting a sign of the metastability of brain dynamics.
Fagerholm et al., 2015	18 healthy adults	EEG-fMRI during rest and a visuomotor cognitive task	Power-law estimation*, shape analysis	• Study examined combined EEG and fMRI in healthy volunteers during rest and cognitive task. Resting-state EEG cascades were associated with approximate power-law distribution, while task state was associated with subcritical dynamics. Decreased response times during the cognitive task were associated with better approximation of a power-law form of cascade distribution. Findings suggest that resting-state was associated with near-critical dynamics while focused cognitive state induced subcritical dynamics with a lower dynamic range to reduce interference with task (i.e. promoting task performance).
Euler et al., 2016	54 healthy adults	EEG at rest and during a working memory task	DFA	• Study finds evidence of inverse relation between theta band LRTC and working memory performance−higher scaling exponent was related to poorer cognitive performance. Authors suggest that since elevated LRTC have been noted in epilepsy, increases in LRTC are not always beneficial.
Cohen, 2016	21 healthy adults	EEG during action adjustment task	Demeaned fluctuation analysis (DMA)	• Real-time error correction has been correlated to an idiosyncratic electrophysiological signature called midfrontal theta. This study found that midfrontal theta is a transient but non-phase-locked response modulated by task performance over three time scales, including scale-free-like fluctuations over many 10 s. The phasic midfrontal theta brain response to errors or error corrections is modulated by slow fluctuations in criticality.
Simola et al., 2017	27 healthy adults	Response times during a Go/NoGo task	Autocorrelation, spectral density, DFA	• Response time fluctuations in the Go/NoGO task exhibited a power law frequency scaling, autocorrelations and LRTCs, with LRTC scaling exponents negatively correlated with the commission error rates. Finding suggested that LRTCs co-exist with cognitive flexibility which is in line with the criticality hypothesis. LRTC scaling exponents were uncorrelated to the mean response time (MRT), suggesting that performance variables derive from distinct processes than brain criticality. Understanding the individual variation in scale-free behavioral dynamics may improve utility of neuropsychiatric assessment in ADHD.
Irrmischer et al., 2018a	28 meditation-trained healthy adults, 21 meditation-naïve healthy adults	EEG during eyes-closed rest and meditation	Spectral density, DFA	• Study evaluated EEG from meditation practitioners and meditation-naive participants from independent labs. In practitioners, but not in controls, meditation strongly suppressed LRTC of oscillations relative to eyes-closed rest, across all frequency bands and scalp locations. Sustained practice led to reduction in LRTC during meditation after 1 year of additional training. Practice also impacted normal waking brain dynamics as reflected in increased LRTC during eyes-closed rest state, indicating an alteration beyond merely meditation. Authors suggested that meditation-induced release of GABA may lead to subcritical regime.
Irrmischer et al., 2018b	57 healthy adults	EEG during eyes-open, eyes-closed, and temporal expectancy task	Spectral density, DFA	• High levels of alpha band LRTC in sensorimotor region during rest predicted good reaction-time performance in attention task. During task execution, fast reaction times were associated with high-amplitude beta and gamma oscillations with low LRTC. • Authors hypothesize that focus and attention move the neural system from near-criticality optimized for environmental and internal demands, to a state of reduced input propagation but increased attentional stability, leading to suppression of LRTC.
Jia et al., 2018	35 children with ASD (ages 4−9 years old), 31 age- and gender-matched neurotypical children	Functional near-infrared spectroscopy (fNIRS) while watching a cartoon	DFA	• The hemoglobin concentration signals (i.e., oxy-Hb and deoxy-Hb) of young children with ASD and normal children were recorded via fNIRS while watching a cartoon. DFA exponents of young children with ASD were significantly smaller over left temporal region for oxy-Hb signal, and over bilateral temporo-occipital regions for deoxy-Hb signals, indicating a shift-to-random-ness of brain oscillations in children with ASD. Testing the relationship between age and DFA exponents revealed that this association could be modulated by autism. • Studying the temporal structure of brain activity via fNIRS technique may provide physiological indicators for autism. Authors speculated about a connection with SOC, though functional significance of DFA exponent is unclear. LRTC could play a role in evaluating disease progression in ASD.
Bongers et al., 2019	22 healthy university students	EEG during computerized learning and resting-state	Irregular Resampling Auto-spectral Analysis (IRASA), power-law estimation	• Study identified power law exponent of fractal signal during continuous EEG of computerized chemistry learning. Mixed power increase of broadband frequencies, which reflected an overall increase in fractal power, was seen during learning. A low power law exponent with increased band power of the fractal component seemed to correlate to high learning gains.
Kwok et al., 2019	41 healthy children (ages 4−6)	High-density EEG during eyes-open and eyes-closed	Spectral density, DFA	• Study used resting state EEG of children with typical development to explore relation between alpha (7−10 hz) oscillations and oral language ability. Higher language scores were correlated with lower alpha power and increased temporal correlations. Findings further demonstrated existence of critical state dynamics as important for language acquisition.
Ezaki et al., 2020	138 healthy adults	Resting-state fMRI; IQ scores	Correlation between spin-glass susceptibility and performance IQ score	• Study added support to criticality hypothesis by showing moderate but clear correlation between IQ scores and distance from criticality at an individual level using dynamic fMRI signals. A model of criticality using spin glasses was compared to data from healthy adults with a range of fluid intelligence IQs. Human fMRI data was found to be within paramagnetic phase close to the boundary with the spin-glass (SG) phase if using the framework of the Ising model. High fluid intelligence was associated with proximity to boundary between paramagnetic and SG phases. SG phase yields chaotic dynamics in spin systems, consistent with idea of enhanced computational performance “at the edge of chaos.”
Ouyang et al., 2020	210 healthy adults	EEG during eyes-open, eyes-closed resting-state, and object recognition task	Structural equation modeling; fitting oscillations and one-over-f (FOOOF) methodology; IRASA	• The goal of this study was to investigate how individual differences in cognitive processing speeds could be predicted by the power spectrum of resting-state EEG signals. Alpha oscillations were not significantly associated with cognitive processing speed once the *1/f* noise was eliminated by SEM. Variation in *1/f* was revealed to robustly predict cognitive processing speed in eyes open and eyes closed. Slope of the power law decaying function was most predictive of between-person processing speed.

Asterisk (*) represents power-law estimations that meet criteria equivalent to or more stringent than Clauset et al. (2009). ASD, autism spectrum disorder; BOLD, blood oxygenation level-dependent; CDF, cumulative distribution function.

In the area of cognition, researchers have wondered whether criticality plays a role in the brain’s response to increasing cognitive loads. In a study of healthy adults undergoing working memory and response tasks, Altamura et al. (2012) found power-law-like behavior in the upper tails of the cumulative distribution of response times. One possible interpretation of this result is the emergence of scale-free behavior in response to increased cognitive loads. In other words, increased cognitive load shifts random behavior toward scale-free behavior near a critical point. Another study of healthy adults undergoing a cognitive task also showed a power-law scaling of response time fluctuations (Simola et al., 2017). Furthermore, Simola et al. (2017) found autocorrelations and LRTC of response time fluctuations, with the LRTC scaling exponents correlating negatively with error rates and positively with executive function testing scores. As the authors pointed out, LRTC can originate in the setting of criticality but also exist in systems with a slow decay of memory. The negative correlation of LRTC scaling exponent with error rates suggests increased cognitive flexibility, which is more consistent with criticality than with a system that operates with slowlydecaying memory. Without criticality, LRTC would decrease cognitive flexibility because long-memory would not allow reconfigurations. Patients with ADHD are known to show increased response time variability in these kinds of tasks. However, in this study, the LRTC scaling exponents were not correlated to response time variability or mean response time, implying that another brain process may be involved in ADHD. Studies of behavioral dynamics (e.g., response times on tasks) in ADHD patients compared to neurotypical patients may grant further insights into the role of scale-free, critical dynamics in ADHD.

But while the behavioral response (i.e., response times) to increased cognitive load may be power-law-like, studies of the electrical brain response to increased cognitive load show more mixed results. A MEG study of neurotypical children and children with high-functioning autism undergoing executive function tasks demonstrated a decrease in power-law scaling of phase synchrony as cognitive tasks became more difficult, i.e., increased cognitive load (Tinker and Velazquez, 2014). Power-law distributions were uncommon in this study in both autistic and neurotypical children and were more likely to co-exist with other distributions (e.g., exponential). These results suggest that metastable, rather than purely critical, dynamics are at play in the brain’s response to increased cognitive loads (Bressler and Kelso, 2001; Deco and Jirsa, 2012). Along similar lines, an EEG study of healthy adults during a working memory task found an inverse correlation between LRTC in the theta frequency range and memory performance (Euler et al., 2016). This result is perhaps the opposite of what one might have predicted from work that had found indications of criticality in behavioral dynamics (Altamura et al., 2012; Simola et al., 2017).

But not all brain studies of human cognition have argued against the existence of critical dynamics. An EEG study of 210 neurotypical adults undergoing an object recognition task showed that variation in *1/f* noise was a robust predictor of cognitive processing speed (Ouyang et al., 2020). Moreover, the power-law exponent of the *1/f* noise was most predictive of person-to-person processing speeds. While there are many non-critical sources of *1/f* noise, this study harmonizes well with the theoretical literature on criticality’s optimization of information transmission. In an EEG study of healthy adults undergoing an action adjustment task, midfrontal theta – an electrical signature known to correlate with real-time error correction (Cavanagh and Frank, 2014) – displayed scale-free-like fluctuations over durations of up to tens of seconds (Cohen, 2016). The author suggested that fluctuations may modulate the midfrontal theta brain response to errors at a critical point (Cohen, 2016). In summary, evidence from both brain dynamics and behavioral dynamics points to some aspects of criticality in human cognition (Altamura et al., 2012; Cohen, 2016; Simola et al., 2017; Ouyang et al., 2020) while other studies do not (Tinker and Velazquez, 2014; Euler et al., 2016).

Studies of human attention have centered on the roles of criticality in meditation and its possible applications to ADHD, as mentioned earlier. There are many forms of meditation, and a commonly practiced form, called focused attention (FA) meditation, helps its practitioners improve their ability to focus (e.g., on their breath, on their bodily sensations). An EEG study of FA meditation-trained subjects compared to meditation-naïve subjects found that FA meditation in practitioners led to strong suppression of LRTC in oscillations across all frequency bands and electrodes, compared to the eyes-closed state (Irrmischer et al., 2018a). Meditation-naïve subjects did not show this suppression of LRTC. A year’s worth of meditation practice led to more permanent changes since FA meditation practitioners had increased LRTC during eyes-closed rest state when they underwent EEG a year later. The authors hypothesized that meditation-related release of GABA might be contributing to excess inhibition and a subcritical regime as seen by decreased DFA exponent (i.e., LRTC suppression). In a similar EEG study of healthy subjects, high levels of alpha frequency LRTC in the sensorimotor region of the brain during rest predicted strong reaction-time performance in an attention task (Irrmischer et al., 2018b). During the execution of the task, on the other hand, suppressed LRTC in beta and gamma frequencies was associated with fast reaction-time performance. This study complements the results obtained in an earlier EEG-fMRI study of healthy adults during a visuomotor task and at rest. It found that the resting-state is associated with near-critical dynamics, while focused cognitive tasks induce subcritical dynamics that may reduce interference with the task at hand (Fagerholm et al., 2015). The upshot of these three studies is that focused attention – whether meditation-related (Irrmischer et al., 2018a) or visual attention (Fagerholm et al., 2015; Irrmischer et al., 2018b) – is associated with a reduction in criticality fluctuations. Generalized human attention at rest, on the other hand, balances a certain degree of focus with the ability to respond quickly to both internal and external stimuli. That balance should theoretically be optimal near criticality.

A handful of studies have examined the role of criticality in human learning, both in children and in adults. A MEG study of children with reading difficulties showed decreased temporal correlations in the left temporoparietal region compared to age and IQ matched children without reading difficulties (Dimitriadis et al., 2013). While this study examined subjects at rest instead of in a reading activity, its results dovetail well with previous studies that showed aberrant cortical activation in left posterior and temporal regions in children with severe reading difficulty during a reading assignment (Hoeft et al., 2007; Cao et al., 2008). Both groups of children exhibited scale-free global network connectivity, suggesting that local, rather than global, decreases in LRTC may be involved in reading difficulties and dyslexia. A high-density EEG study of neurotypical children found that lower alpha frequency power and increased LRTC correlated positively with language scores (Kwok et al., 2019). This study confirmed the findings of Dimitriadis et al. (2013) that critical state dynamics are important for language acquisition.

A recent resting-state fMRI study of neurotypical adults with a range of IQ scores found that high fluid intelligence is associated with proximity to a critical phase transition in a spin-glass model (Ezaki et al., 2020). This finding was consistent with previous work suggesting near-criticality as perhaps optimal for learning (Gisiger et al., 2014). From an EEG study of healthy university students learning organic chemistry, it is also known that a lower power-law exponent of the fractal component of EEG signals correlates with higher learning gains during a computerized learning task under EEG (Bongers et al., 2019). While this latter study makes no claims regarding criticality, one can infer that the scale-free behavior of neurons matters for the acquisition of complex new knowledge.

Only a few studies have examined the role of criticality-related markers in autism. A resting-state fMRI study of adult males with autism spectrum disorders, including high-functioning autism, detected a small but significant decrease in the Hurst exponent compared to controls in brain regions implicated in autism by previous neuroimaging studies (Lai et al., 2010). This decrease in the Hurst exponent indicates a shift toward randomness of fluctuations in blood oxygen level-dependent (BOLD) signals in the brain impacted by autism. Though the interpretation of the Hurst exponent in BOLD signals is challenging because of the unclear roles of blood supply and neural circuits among many others, the use of fractal scaling can serve as a barometer of underlying coordination and organization of neural circuits. In this study, the shift toward randomness in BOLD signals may indicate decreased coordination of small-scale circuits, to the disadvantage of larger brain circuitry. This hypothesis harmonizes well with one of many prevailing theories about the origins of autism, namely the local overconnectivity theory (Belmonte et al., 2004; Baron-Cohen and Belmonte, 2005). A more recent study of children with autism spectrum disorders (ASD) used functional near-infrared spectroscopy (fNIRS) to compare LRTC with age and gender-matched neurotypical children (Jia et al., 2018). Consistent with the findings of Lai et al. (2010) this study found that DFA exponents (i.e., LRTC scaling exponents) were significantly smaller over the left temporal region for the oxy-hemoglobin (oxy-Hb) signal in children with ASD compared to controls. DFA exponents were also significantly smaller over both temporo-occipital regions for deoxy-hemoglobin (deoxy-Hb) signals in children with ASD compared to controls. DFA exponents correlated well with age in neurotypical children, consistent with findings discussed previously (Smit et al., 2011). However, DFA exponents of oxy-Hb in the left temporal region correlated negatively with autism symptom severity on a parental questionnaire. This result dovetails nicely with a previouslyidentified negative correlation between cerebral blood flow to the left superior temporal gyrus and Autism Diagnostic Interview-Revised scores (Meresse et al., 2005). While the authors of this fNIRS study suspect that autism may represent a departure from SOC, it seems further neuroimaging studies using criticality-based approaches will be needed before one can safely reach that conclusion.

### Psychiatry

Researchers have turned to criticality-based tools to improve their understanding of common psychiatric conditions like depression, schizophrenia, anxiety, post-traumatic stress disorder (PTSD) (see Table 6). Insights from criticality theory have also helped describe the psychological effects of neurofeedback and psychedelics.

**TABLE 6:** Summary of psychiatry-related criticality literature.

Study	Study population	Modality	Analysis	Main findings
Linkenkaer-Hansen et al., 2005	12 adults with MDD, 10 age-matched healthy controls	MEG during eyes-closed resting state	DFA, linear correlation of DFA exponent to Hamilton Depression Rating Scale	• This study recorded MEG data from patients with MDD compared to healthy controls during eyes-closed wakeful rest and quantified LRTC in amplitude fluctuations of different frequency bands. Temporal correlations in theta band were absent in the 5−100 s time range in patients but were prominent in controls. The magnitude of temporal correlations over left temporo-central region predicted severity of depression in patients. LRTCs in theta oscillations are a salient characteristic of healthy human brain and may have diagnostic potential in psychiatric disorders.
Lee et al., 2007	11 unmedicated adults with MDD per DSM-IV, 11 non-depressed age-matched controls	EEG during resting state	DFA	• Study compared LRTC in depressed subjects compared to controls. Study found a significant linear correlation between severity of depression and scaling exponent over most channels. There was slower decay of LRTC and persistence of LRTC in depressed patients associated with severity of depression over most cortical areas.
Slezin et al., 2007	33 patients with paranoid schizophrenia, schizotypal disorder, schizoaffective disorder per ICD-10, 23 healthy controls	EEG during resting state	Spectral density, power-law estimation	• Study applied multifractal analysis of *1/f* EEG rhythm fluctuations in patients with schizophrenia-related disorders compared to controls. Study found increased instability and randomness of alpha rhythm in the F4 electrodes in schizophrenia-related disorders. Theta rhythm, on the contrary, showed increased stability, regularity, and decreased complexity compared to normal/healthy controls in the same disorders.
Leistedt et al., 2007b	10 untreated inpatients with an acute episode of MDD per DSM-IV, 14 healthy controls	EEG during sleep	spectral density, DFA	• Major depressive episodes are characterized by modification in correlation structure of sleep EEG time series. Power law exponents were lower but not statistically significant in stage 2 and NREM3-4. These changes in scaling behavior could provide an explanation for why patients with acute depression have sleep fragmentation.
Leistedt et al., 2007a	10 untreated men in full or partial remission from MDD per DSM-IV; 14 healthy controls	EEG during sleep	DFA	• Goal of the study was to investigate the scaling properties of the sleep EEG in remitted depressed men and to see whether history of MDD could significantly alter dynamics of sleep EEG as a “scar marker.” No significant differences were noted between the two groups during sleep. There were no functional sequelae of past history of one or more unipolar MDD episodes on fluctuation properties of sleep EEG. Study argued against “depressive scar hypothesis” in which some permanent residual defect is created by depressive disease. Study also confirmed LRTC in human sleep EEG.
Tolkunov et al., 2010	50 healthy adults without psychiatric history	fMRI during visual stimulus testing	power spectrum scale invariance (PSSI)	• Study examined whether patients with higher levels of trait anxiety would show less efficient regulation of limbic responses using a visual stimulus during fMRI. Significant positive correlations were found between beta frequency for limbic control circuit and trait anxiety. Dysregulated outputs from limbic system in trait anxiety also led to dysregulated inputs to the autonomic nervous system.
Radulescu et al., 2012	9 adults with schizophrenia per DSM-IV, 26 healthy controls	fMRI scan during affect-valent stimulus	Power spectrum scale invariance (PSSI), Poincare maps	• Study hypothesized that paranoid schizophrenia might be result of optimization abnormalities in the prefrontal-limbic circuit that regulates emotion. Patients and controls showed distinct PSSI in the orbitofrontal/medial prefrontal cortex (Brodmann 10). Poincare maps showed less variability in patients compared to controls. PSSI may be useful for psychiatric diagnoses, partly for the spatial localization it affords.
Nikulin et al., 2012	18 adults with schizophrenia, 3 with schizoaffective disorder per DSM-IV, 28 healthy age- and gender-matched controls	high-density EEG during resting state	DFA, cross-frequency correlations	• Study evaluated LRTC in schizophrenia-related disorders compared to healthy controls. LRTCs were significantly decreased in patients with schizophrenia in both alpha and beta frequency ranges. Authors hypothesize that decrease in LRTC arises from increased variability in neuronal activity in patients with schizophrenia. Haloperidol dosing and scaling exponent did not correlate in electrodes or frequency bands, which argues against the effects of anti-psychotics on the noted differences in LRTCs.
Bornas et al., 2013	56 healthy undergraduate students	EEG during eyes-open, eyes-closed resting state; Beck Depression	DFA, linear correlation between questionnaire scores and DFA exponents	• Goal of study was to look for possible differences in LRTC in brain signals from people with different negative emotion regulation strategies, including rumination, that lead to a depressed lifestyle. Study identified linear positive correlations between the scaling exponents of both broad band and theta band oscillations and negative emotion regulation strategies and depression
		inventory and emotion regulation questionnaires		scores. Authors suggested that differences may exist between depressed and non-depressed even before depression manifests, though depressed mental state clearly impacts the degree of correlation.
Tagliazucchi et al., 2014	15 healthy adult subjects	fMRI before, during, and after IV psilocybin and placebo infusions	Variance and total spectral power	• Study aimed to quantify the repertoire of neural states under the influence of psychedelics like psilocybin. Changes in spectral scaling exponents and variance of BOLD signals exclusively affected higher brain systems. Authors found that psilocybin resulted in a larger repertoire of connectivity states at rest than in control conditions, consistent with brain criticality.
Zhigalov et al., 2016	9 healthy adult subjects (ages 18−23 years old)	EEG while resting with eyes-closed during closed-loop and sham NFB sessions	Spectral density, DFA	• Study hypothesized that LRTC could be manipulated by closed-loop NFB stimulation. Over multiple sessions, there emerged a significant difference in LRTCs of α-band oscillations, with LRTCs stronger during NFB than sham. Study served as a proof-of-concept that EEG LRTCs, and thus critical brain dynamics, could be modulated with closed-loop stimulation.
Ros et al., 2014	40 healthy adults; 21 adults with PTSD per DSM-IV, with 30 age- and gender-matched healthy controls	fMRI scan before NFB, EEG during NFB or sham-NFB, and fMRI scan after NFB	DFA	• Study aimed to evaluate possibility of manipulating LRTC in patients suffering from PTSD. Brain areas with low LRTCs in PTSD subjects normalized toward healthier population levels with application of neurofeedback compared to sham. Authors suggest that LRTC changes seen with NFB are due to fluctuations in excitation-inhibition balance.
Gärtner et al., 2017	71 depressed adult patients per DSM-IV, 25 healthy controls	EEG before and after mindfulness training or stress reduction training	DFA	• Study sought to understand whether neural dynamics improved in patients with MDD after psychological treatment. Depressed subjects exhibited stronger LRTC in theta oscillations (4–7 Hz) at baseline compared to controls. Following the psychological interventions both groups exhibited decreased LRTC in the theta band, with marginal numerical differences between the groups. Future of this research area will involve uncovering how psychological interventions effectively reduce LRTCs.
Moran et al., 2019	23 patients with schizophrenia per DSM-IV, 24 education-, handedness-, age-, and gender-matched healthy controls	EEG with eyes-open	DFA, LORETA	• Patients with schizophrenia showed area of significantly reduced beta-band LRTC over bilateral posterior regions compared to controls. Absence of alpha band differences (contrary to Nikulin et al., 2012) could be related to eyes-open EEG used in this study.

*DSM-IV, Diagnostic and Statistical Manual, 4th edition; MDD, major depressive disorder; NFB, neurofeedback.*

Studies of major depressive disorder (MDD) or unipolar depression have mostly relied on the search for temporal correlations (LRTC) using DFA. A small MEG study of patients with MDD and healthy controls found absent LRTC in the theta frequency band in patients with MDD compared to controls (Linkenkaer-Hansen et al., 2005). The study authors suggested that abnormal temporal structure of theta oscillations could reflect an underlying defect in limbic-cortical networks identified in anatomic-functional studies of MDD. Another small EEG study of patients with MDD and healthy controls did not reproduce this finding of absent theta LRTC (Lee et al., 2007). Instead, the study authors found that increased LRTC scaling exponents (i.e., slower decay of LRTC) correlated positively with the severity of depression over most EEG channels. This finding led the authors to speculate that rumination and psychomotor retardation – typical features of MDD – may be responsible for this persistence of LRTC.

Other studies have examined whether depression leads to alterations in LRTC during sleep. A small sleep EEG study of patients with untreated, acute episodes of MDD found a decrease in LRTC scaling exponents in NREM2, NREM3, and NREM4 compared to healthy controls (Leistedt et al., 2007b). However, this decrease was not statistically significant. A similar study by the same research team, this time with patients in remission from MDD, found no differences in LRTC through the stages of sleep (Leistedt et al., 2007a). In addition to demonstrating the existence of LRTC during sleep (see Sleep Medicine section for more details), these results argue against the “depressive scar” hypothesis, according to which depression leads to permanent residual defects.

Researchers have also taken an interest in negative emotional regulation as a precursor for MDD. A large EEG study of non-depressed undergraduate students found a positive linear correlation between LRTC scaling exponents of theta and broad-band frequencies and negative emotional regulation on various questionnaires (Bornas et al., 2013). Their research suggested that negative emotional control may anticipate full-blown MDD by several years. While this study broadly agreed with the findings of Lee et al. (2007), it disagreed with Linkenkaer-Hansen et al.’s (2005) conclusions with regards to the magnitude of scaling exponents with worsening rumination. A more recent, large EEG study found that, at baseline, patients with MDD have higher LRTC scaling exponents in the theta frequency range than healthy controls (Gärtner et al., 2017). After intervention with either mindfulness training or stress reduction training, both groups experienced a reduction in the strength of LRTC. This latter result suggests a new approach for examining the physiological mechanism by which psychotherapeutic interventions improve depressive symptoms. Given the inconsistencies in findings, more extensive studies will help clarify the nature of the LRTC in the various frequency bands and the multiple stages of depression (acute, treated, etc…). Future studies could assess whether LRTC changes significantly after initiation of first-line pharmacological intervention for MDD. Other criticality-based metrics could be useful to characterize the nature of brain dynamics in MDD as either sub-critical or, less likely, super-critical.

Criticality-based studies of schizophrenia are few but have opened up new horizons. An EEG study of around 30 patients with schizophrenia and schizoaffective disorders used a fractal analysis of *1/f* EEG rhythm fluctuations to try to distinguish the brain dynamics of schizophrenia from that of healthy controls (Slezin et al., 2007). A frontal electrode (F4) exhibited increased instability and randomness in the alpha band for patients with schizophrenia and schizoaffective disorders. The theta band, on the other hand, exhibited increased stability and decreased complexity in patients with schizophrenia compared to healthy controls. A subsequent fMRI study of patients with schizophrenia compared to healthy controls (Radulescu et al., 2012) also identified decreased complexity of brain dynamics, but different from that of Slezin et al’s. (2007)study. In Radulescu et al’s. (2012) study, a sophisticated analysis pipeline, including power spectrum scale invariance (PSSI) and Poincare maps (a measure of signal variance), found that signals coming from Brodmann area 10 (BA10) exhibited lower power-law exponents in schizophrenic patients than in healthy controls. In other words, schizophrenic patients displayed white (Gaussian or random) noise in this brain region, compared to pink (1/f) noise in healthy patients. This finding is consistent with previous research documenting the role of BA10 in executive function, working memory, and emotion regulation (Radulescu et al., 2012). This transition from pink noise to white noise in schizophrenia suggests, from the perspective of criticality, a loss of responsiveness to stimuli.

While the studies mentioned above took a fractal and power-law approach to signal analysis, others have taken the route of studying LRTC in schizophrenia. Their results complement and refine those obtained using fractal approaches. A high-density EEG study of LRTC in adults with schizophrenia and schizoaffective disorders found strong attenuation of LRTC scaling exponents in alpha and beta frequency bands in patients compared to healthy controls (Nikulin et al., 2012). While Slezin et al. (2007) did not study the beta frequency range, they did observe increased complexity in the alpha range, which is globally consistent with the findings in this study. These attenuated scaling exponents indicate decreased temporal correlation and a decrease in temporal precision of neuronal firing compared to healthy subjects. This interpretation fits well with at least two theories for the origins of schizophrenia, namely that of excessive neuronal noise and variability (Rolls et al., 2008) and the “disconnection hypothesis” of dysfunctional relationships between neural networks (Friston et al., 2016). A follow-up study found similar results (Moran et al., 2019). In this high-density EEG and standardized low-resolution brain electromagnetic tomography (sLORETA) study of patients with schizophrenia compared to healthy controls, there was a significant reduction in LRTC in the beta-band over the bilateral posterior regions in patients compared to healthy controls. This study found no differences in the alpha frequency between patients and controls. However, the study authors suggested that the eyes-open state of their study (compared to the eyes-closed, resting state of Nikulin et al., 2012) may have eliminated an underlying difference in that frequency range. More importantly, both studies confirm the importance of LRTC as markers of network instability in schizophrenia.

A much smaller number of studies have examined the role of neurofeedback (NFB) as a treatment modality for psychiatric disorders. A small study of healthy adults randomized to either closed-loop NFB or sham NFB found that, after three sessions, LRTC in the alpha frequency band was stronger in the NFB group compared to the sham (Zhigalov et al., 2016). This difference was statistically significant and did not involve any statistically significant topographical changes in alpha power. The study provided proof-of-concept that closed-loop NFB can restore critical brain dynamics by altering the excitation-inhibition balance in psychiatric disorders with decreased LRTC. A larger EEG and fMRI study of healthy patients confirmed this improvement in LRTC after closed-loop NFB compared to sham. The study also involved a population of patients with PTSD, whose LRTC improved with closed-loop NFB (Ros et al., 2014). The study authors speculated that NFB leads to stronger excitation with an associated increase in temporal correlation and symptomatic improvement. While it is unclear whether NFB can help in disorders characterized by super-criticality (e.g., seizures), NFB has already shown benefit in several disorders characterized to some degree by sub-criticality, including schizophrenia (Surmeli et al., 2012) and major depression (Escolano et al., 2014).

A few criticality-based studies have taken an interest in the role of psychedelics in treating mental illnesses. Researchers have focused on psilocybin, the active ingredient in “magic mushrooms,” and lysergic acid diethylamine (LSD). In an fMRI study of healthy patients receiving psilocybin versus a placebo, study authors found an increased variance in BOLD signals in the hippocampi of psychedelic recipients (Tagliazucchi et al., 2014). The increased variance implies increased synchronization, consistent with a super-critical state of brain dynamics. This super-critical state could account for the hyper-associative cognition characteristic of psychedelics (Carhart-Harris et al., 2014; Carhart-Harris, 2018). While this review limits itself to non-connectomic data, the works of Atasoy et al. (2017, 2018) deserve mention here. Using a unique connectome-harmonic decomposition approach, her research team has found in several studies that infusions of psychedelics “push” brain dynamics out of baseline sub-criticality toward criticality (Atasoy et al., 2017, 2018). More and more studies are finding that psychedelics are helpful adjuncts to psychotherapy (Atasoy et al., 2017). A psychedelic-induced transition out of a sub-critical disorder like depression and closer to criticality may account for this benefit.

### Sleep Medicine

Sleep medicine deals with human sleep and its associated disorders, including insomnia, sleep apnea, and narcolepsy. There are multiple stages of sleep, including rapid eye movement (REM), non-rapid eye movement (NREM), which is further divided into light sleep (NREM1 and NREM2) and deep sleep (NREM3 and NREM4). A typical night’s sleep runs through several cycles of each of these sleep stages. The brain criticality literature centers on two key areas of sleep medicine – characterization of sleep stages and the physiology of sleeping disorders.

Researchers have tried improving the classification of sleep stages with the help of criticality-based metrics. The results of these studies seem, for now, largely inconsistent. An EEG study of healthy patients found LRTC in brain oscillations in both the open-eye and closed-eye states (Nikulin and Brismar, 2004). LRTC was more consistent, however, in the closed-eye state, with a significant difference in the scaling exponent between closed and open eye conditions in the beta frequency range. Building on this initial result for LRTC, an EEG study of healthy patients examined the ability of various fractal and spectral metrics to distinguish REM, NREM2, and NREM4 (Weiss et al., 2011). The authors found that the multifractal metric was superior to other metrics for sleep stage classification. From wakefulness to deep sleep, multifractality decreased while monofractality (e.g., the Hurst exponent) increased. The loss of multifractality and disruptions in the *1/f* noise in the deep sleep stages suggested that sleep-specific brain rhythms (e.g., sleep spindles) disrupt the day-time self-similarity. Previous studies had shown an increase, not a decrease, in multifractality in deepening sleep (Ma et al., 2006). These differing results suggest that additional studies of multifractality are needed.

The increase in mono-fractality that accompanied deep sleep in the study by Weiss et al. (2011) suggested increased long-memory (LRTC) in sleep, similar to the increase in scaling exponent noted in the closed eye state by Nikulin and Brismar (2004). Subsequent studies, however, have produced results that seem to contradict these findings. Using EEG-fMRI data from healthy adults in NREM sleep, Tagliazucchi et al. (2013) found that a breakdown in LRTC happened as patients moved into the deeper sleep stages. Specifically, the Hurst exponent decreased in the default-mode network (DMN) and attention centers of the brain during NREM2 (Tagliazucchi et al., 2013). Two studies of polysomnogram data from a different cohort of healthy adults found a similar result (Allegrini et al., 2013, 2015). The monofractal metric (i.e., the Hurst exponent) decreased from ∼0.75 during wakefulness and REM, to ∼0.5 in NREM3 and NREM4. Recall that a Hurst exponent of 0.5 indicates random, white noise, while 0.75 suggests a moderate amount of positive correlation. This finding suggested the existence of a breakdown in LRTC when entering deep sleep. Complicating matters further is that studies showing decreases in LRTC during sleep did not control for changes in signal power that naturally occur during sleep (Meisel et al., 2017). More studies on the existence and changes of LRTC during sleep, taking into account fluctuations in signal power, are needed if these are to become useful in sleep stage classification or in understanding sleep microarchitecture.

There are also conflicting results in the literature when it comes to power-law distributions of various observables during sleep. In an EEG study, researchers noted that subjects in normal states of wakefulness exhibited a power-law distribution of coherence potentials and phase-locking intervals (PLI)(Meisel et al., 2013). Also, their EEG signals produced a branching parameter near one, which suggests a system at or near criticality (Yang et al., 2012; Meisel et al., 2013). As patients were kept awake longer and deprived of sleep, the distributions of coherence potentials and PLIs developed larger tails along with an increase in the branching parameter, a decrease in the variability of synchronization, and an increase in mean synchronization – all suggestive of a supercritical state. The authors suggested that prolonged wakefulness leads to a supercritical state through excess excitability of neurons (Shew et al., 2009), as is also thought to occur in epilepsy. Meisel et al. (2013) proposed that, during restorative sleep, a decrease in synaptic strength should lead to a shift away from super-criticality, back toward a critical regime. This view is in line with a similar theory that sleep is designed to restore brain dynamics to a slightly subcritical or near-critical regime to avoid the risk of run-away excitation in a supercritical state (Pearlmutter and Houghton, 2009, 2013).

An ECoG study of patients during sleep revealed power-law distributions of neuronal avalanches in local field potentials (LFPs), but with different exponents depending on the sleep stage (Priesemann et al., 2013). After making adjustments for the fact that LFP amplitude naturally increases from wakefulness to deep sleep, the study authors found that slow-wave sleep (SWS or NREM3) displayed large avalanches. Wakefulness and REM sleep, on the other hand, displayed intermediate and small avalanches, respectively. Because the study found a branching parameter less than one during wakefulness, the authors concluded that the awake human brain at rest is in a subcritical state rather than in a critical state as had been previously argued (Meisel et al., 2013). The authors suggest that brain dynamics shift closer to criticality during SWS and then back into sub-criticality during REM sleep (Priesemann et al., 2013). Their data and additional computational modeling found that small changes in effective synaptic strength could tune the brain closer to a critical state during SWS or closer to a subcritical state with fragmented dynamics during REM. Of note, this study did not find power-law distributions under the more stringent criteria of Clauset et al. (2009). Moreover, a small study of power-law distributions of cortical spikes and LFPs under awake, SWS, and REM sleep conditions could not confirm a power-law distribution of those brain signals using the Clauset et al. (2009) criteria (Dehghani et al., 2012).

While the study of Dehghani et al. (2012) casts doubt on these power-law findings, a recent study of EEG-fMRI data in healthy patients during sleep suggests that critical dynamics may be involved nonetheless (Bocaccio et al., 2019). A power-law distribution of co-activated connected clusters (voxel groups) from fMRI data during sleep was confirmed using the Clauset et al. (2009) criteria. As in Priesemann et al. (2013), this study found larger neuronal avalanches in NREM compared to REM or wakefulness. Moreover, each particular sleep stage impacted the power-law exponent significantly – a result that was independent of spatial coarse-graining of the fMRI data and which could not be accounted for by the presence of evoked neural bistabilities (e.g., K complexes).

All in all, while there are discrepancies in the results obtained from studies in this area, one can be optimistic about the role of criticality-based metrics in refining the understanding of sleep stages and their classification. Research into the role of LRTC (with adjustments made for signal power during sleep), branching parameters, power-law distributions of various metrics (coherence potential, PLI, LFP), mean synchronization, and other criticality parameters should lead to robust classifiers of sleep stages.

The conceptual framework of criticality is also helping researchers better understand various sleep pathologies (Iyer, 2018). Many animal and computational models show that critical dynamics play a central role in sleep stage transitions (Comte et al., 2006; Wang et al., 2019; Lombardi et al., 2020). Human studies are also beginning to capture this essential role by studying various sleep disorders. In the previouslymentioned EEG study of sleep deprivation (Meisel et al., 2013), there was a progressive decline in markers of criticality and an increase in markers of supercriticality as the sleep deprivation worsened. This finding supports the idea that sleep restores healthy brain function by bringing dynamics back toward criticality. This study was followed up by an evaluation of LRTC in the same cohort under similar conditions of sleep deprivation (Meisel et al., 2017). After controlling for signal amplitude changes, LRTC strength declined as sleep deprivation progressed, consistent with the view that sleep restores brain criticality.

In contrast with forced sleep deprivation, insomnia disorder (ID) leads to chronic sleep deprivation despite the patient’s attempts to fall asleep. In a study of patients with ID, there were no statistically significant differences in the LRTC during eyes-open and eyes-closed testing between patients and their age and sex-matched controls (Colombo et al., 2016). However, patients who subjectively reported lower quality sleep had increased LRTC during the eyes-open state. The study authors suggested that an increased excitation-inhibition ratio during wakefulness may translate into similar excitation during sleep, leading to lower sleep quality. Similar processes seem to be at work in obstructive sleep apnea (OSA), one of the most common sleep disorders. A polysomnogram study of patients with OSA and healthy controls found a power-law distribution of wakefulness durations during sleep in both groups (Lo et al., 2013). But the study did find a statistically significant difference between the power-law exponents. This finding reinforces the prospect that power-law exponents, perhaps in combination with other established metrics like the apnea-hypopnea index, could prove to be a helpful marker of diagnosis and monitoring of OSA. In the future, a study of LRTC in patients with OSA and ID compared to healthy patients in sleep could help determine whether the prevalent theory of sleep as a safety margin from supercriticality is correct. More broadly, the theory of brain criticality is likely to offer a different but complementary perspective on many sleep disorders (including narcolepsy, restless legs syndrome, and circadian disorders). See Table 7 for a summary of findings from the sleep-related criticality literature.

**TABLE 7:** Summary of sleep-related criticality literature.

Study	Study population	Modality	Analysis	Main findings
Nikulin and Brismar, 2004	12 healthy adults	EEG with eyes-open and eyes-closed	DFA	• LRTC in alpha oscillations were not changed significantly by wakefulness level while beta oscillation scaling exponent significantly increased in the closed eye condition. Increased synaptic activity associated with arousal/wakefulness may interfere with dynamics of LRTC. Study confirmed existence of LRTC in both awake and closed eyes but more consistently in closed eye state and may be reflective of underlying SOC.
Weiss et al., 2011	22 healthy adults	EEG during REM, NREM2, NREM4 sleep	Hurst exponent, power spectral measures	• Study assessed various metrics of sleep EEG including monofractal, multifractal, and spectral power measures. Sleep stage discrimination with multifractal measure was superior to relative band powers, spectral edge frequency, or Hurst exponent. • Study found higher H exponent, DFA exponent, and fractal exponent in deep sleep, while multifractal measure was decreased. These findings indicate a decrease of multifractality and an increase in long memory in deep sleep.
Dehghani et al., 2012	2 adult patients with intractable epilepsy	iEEG from temporal gyrus in awake state, REM, and slow-wave sleep	Power-law estimation*	• Study investigated power-law distribution of neuronal avalanches (spikes) from iEEG data. Neuronal avalanches (spikes) did not clearly follow power-law in awake, SWS, or REM states and instead followed closer to exponential distribution. Positive and negative LFPs followed apparent power laws with log-log analysis but closer examination with CDF-based testing did not confirm power law and favored double exponential distribution. In cases where power laws were seen with log-log analysis, exponents were too high for SOC systems. • These results contradict those of prior studies (Petermann et al., 2009; Ribeiro et al., 2010) and perhaps could be harmonized with prior results by taking into account recording methods or volume conduction effects.
Meisel et al., 2013	8 healthy adults	EEG during 40 h of sustained wakefulness	Power-law estimation*, branching parameter, spectral density	• Study evaluated evolution of criticality parameters during prolonged wakefulness. At the start of sleep deprivation, coherence potentials were organized as neuronal avalanches in space and time with power law −3/2 and branching parameter 1.17, both of which suggest a system near criticality. With increased duration of wakefulness, size distributions of coherence potentials and PLIs developed larger tails, an increase in branching parameter, and an increase in mean synchronization while variability of synchronization decreased. • These findings suggested that, during sustained wakefulness, the neural networks move from a critical to a supercritical state, perhaps as a result of increased excitation and decreased inhibition (Shew et al., 2009; Yang et al., 2012). Sleep might serve to reorganize network dynamics to critical state in order to assure optimal computational capabilities while awake.
Priesemann et al., 2013	5 adults with refractory epilepsy	iEEG (depth electrode)	Power-law estimation*, branching parameter	• Neuronal avalanches were recorded from intracranial depth electrodes in 5 epilepsy patients over two nights through all sleep stages. Avalanches were described by power laws in all cases but with different dynamics depending on sleep stages. SWS showed the largest avalanches, wakefulness showed intermediate-sized, while REM showed smallest. Differences in avalanche distributions implied that not all vigilance states could be derived from SOC. • Modeling suggested that human brain operates within subcritical regime, near criticality where differences between vigilance states can be mediated by small changes in effective synaptic strength which allow the brain to tune closer to criticality (SWS) or farther away (REM). SWS showed increased correlations between cortical areas due to increased criticality, while REM sleep showed more fragmented dynamics than SWS and wakefulness.
Lo et al., 2013	48 healthy adults and 48 age-matched adults with obstructive sleep apnea (OSA)	Polysomnogram recorded for 2 consecutive nights	Probability transfer matrix, power-law estimation	• Study found a power law distribution of wake and arousal durations in sleep using log-log analysis. Power-law exponents were different between patients with OSA and healthy controls. • Using novel probability transfer matrix and SOC, authors revealed sleep transition pathways that could be reduced to two basic and independent transition paths. Study also found that sleep micro-architecture at scales from seconds to minutes exhibits a non-equilibrium behavior reminiscent of critical systems.
Tagliazucchi et al., 2013	63 healthy adults	fMRI and EEG during NREM sleep	DFA	• Study hypothesized that breakdown of LRTC would occur during descent into deep sleep. Authors found that Hurst exponent decreased during N2
				sleep confined to DMN and attention networks. Study also discovered that autocorrelation in fronto-parietal areas diminish from wakefulness to deep sleep.
Allegrini et al., 2013	29 healthy adults	Polysomnogram	Random walks, DFA, fractal intermittency	• Study hypothesized that a renewal point process describing fractal intermittency could be a correlate of consciousness. Fractal intermittency can be seen in EEG data by sequence of global rapid transition processes (RTP) with power law distribution of waiting times. During sleep, Hurst exponent switched from 0.75 in wake and REM phases to 0.5 in deep sleep, suggesting fractal intermittency in wake and REM but short-time correlations in SWS.
Allegrini et al., 2015	29 healthy adults	Polysomnogram	Random walks, DFA, fractal intermittency	• Study evaluated fractal intermittency (see Allegrini et al., 2013) during sleep. While critical avalanches remained unchanged, there was a breakdown in intermittency and functional connectivity during shallow and deep NREM sleep. The authors provided a theory for fragmentation-induced intermittency breakdown. The possible role of critical avalanches in dreamless sleep is to provide rapid recovery of consciousness if stimuli arouse the person out of sleep.
Colombo et al., 2016	52 adults with insomnia disorder (ID), 42 age- and sex-matched controls	High-density EEG with eyes-open (EO) and eyes-closed (EC)	DFA	• There were no differences in DFA exponents between ID and controls in any frequency bands during EO or EC. However, during EO, individuals with worse sleep quality had stronger LRTC, suggesting that subjective insomnia complaints involve distinct processes in people with ID and controls. However, the measurement of insomnia severity was based on subjective report, not polysomnography. Future studies should examine polysomnographic data as well as examine frequency (rather than amplitude) fluctuations.
Meisel et al., 2017	8 healthy adults	EEG during 40 h of sustained wakefulness	DFA, autocorrelation function, spectral density	• Study evaluated LRTCs in resting state human EEG during 40-h sleep deprivation experiment. LRTCs declined as sleep deprivation progressed, even when taking into account changes in signal power. LRTCs naturally emerged in vicinity of critical state. Authors argued that the increased LRTCs seen in insomnia patients (Colombo et al., 2016) could be due to signal power changes associated with worse sleep quality.
Bocaccio et al., 2019	18 healthy adults	fMRI and EEG during wakefulness and all sleep stages	Power-law estimation*	• Study observed scale-free hierarchy of co-activated connected clusters using point-process transformation of fMRI data recorded during wake and NREM sleep. Sleep stage had significant impact on scaling parameter of power law, which was robust to spatial coarse-graining, alternative statistical models, and disappearing with phase shuffling of fMRI time series. These findings suggest the existence of larger clusters or avalanches during N2 sleep. Criticality may help with the “pretty hard problem of consciousness” by offering metrics that behave one way in conscious states and differently in another.

Asterisk (*) represents power-law estimations that meet criteria equivalent to or more stringent than Clauset et al. (2009). SWS, slow-wave sleep; REM, rapid eye-movement; NREM, non-rapid eye-movement; PLI, phase-locking interval; DMN, default mode network.

## Conclusion

This scoping review surveyed the brain criticality literature, focusing on seven major domains of clinical application. Wherever possible, an effort was made to emphasize areas of future research for those interested in pursuing a “critical approach” to these clinical questions. In this concluding section, controversies that continue to be problematic for the field of brain criticality as a whole are addressed.

Brain criticality is both an established area of neuroscience research and yet remains controversial in several regards (Wilting and Priesemann, 2019).

1. Diverse and inconsistent uses of the terms “critical” and “criticality” have led to confusion. In this review, criticality has mostly referred to avalanche dynamics that behave at the limit between stability and instability. But other variants of criticality exist (Wilting and Priesemann, 2019). These include criticality between ordered and chaotic phases called the “edge of chaos” (Boedecker et al., 2012), criticality between synchrony and asynchrony (Botcharova et al., 2014), and multiple paradigms for the time-evolution of a critical phase transition as in extended criticality, intermittent criticality, and self-organized criticality (Saleur et al., 1996; Huang et al., 1998; Sammis and Smith, 1999; Bowman and Sammis, 2004). These forms of dynamical criticality are also distinct from statistical criticality (Mora and Bialek, 2011; Tkačik et al., 2013). How these all inter-connect is a topic of ongoing research. Rigorously defining these states and enforcing a more consistent vocabulary would allow for better study comparison.
2. Proving the existence of a control parameter has been difficult. Candidates have included synchronization, excitation-inhibition balance, and synaptic conductance (Beggs and Timme, 2012). Feedback processes between these properties may make it difficult to prove that they behave as genuine control parameters in isolation.
3. Many of the publications that lay the groundwork for brain criticality did not have the benefit of a reliable statistical framework for verifying a power-law distribution. While publications since Clauset et al. (2009) have mostly implemented strong statistical testing of power laws, many of the initial papers and even some more recent publications suffer from this deficit. Establishing a standard pipeline of statistical analysis for common datasets (e.g., EEG, MEG, LFP) – a kind of statistical “best practices”−will eliminate a source of confusion and help clarify the clinical situations in which power-law regimes do and do not exist. Moreover, arguing for a mechanism that generates a power-law – whether criticality or another mechanism−may be as important as the discovery of the power-law distribution itself (Stumpf and Porter, 2012).
4. In many publications, the objects of study are defined differently from one paper to the next. Such is the case, for example, with neuronal avalanches (Wilting and Priesemann, 2019) and seizure energy (Worrell et al., 2002; Osorio et al., 2009). While flexibility in definitions may help with making discoveries or adjusting for specific experimental situations, frequent changes in definition make it difficult to compare results, which slows down the progress of the field as a whole.
5. A common strategy, especially since the invention of DFA, is to look for LRTC as an indication of criticality. However, recent research suggests there may be different types of LRTC at play in these various systems and not distinguishing them carefully may be leading to false conclusions about criticality. For example, the discovery of crucial events in the area of turbulence led to the identification of “crucial event LRTC” or CELRTC (Bohara et al., 2018; Culbreth et al., 2019). CELRTC emerges in critical systems, specifically self-organized temporal criticality (SOTC) and is based on a slowly-decaying, non-stationary correlation function (Mahmoodi et al., 2017). CELRTC is distinct from Hurst exponent LRTC (HLRTC) which is based on a slowlydecaying but stationary correlation function, and which may not be indicative of underlying criticality. Future papers relying on detection of LRTC should incorporate this methodology in order to clarify the origin of the long-range correlation.
6. DFA was introduced for the study of non-stationary datasets. Yet there is evidence that this approach may not be adequate for that purpose (Bryce and Sprague, 2012). The introduction of a finite-size effect by DFA, leading to artifact, may have led to spurious results in many publications mentioned in this review. Future research should clarify the relevance of DFA-based methods for the purpose of LRTC detection.

Despite these challenges and controversies, there is also room for optimism. There has been rapid growth in the number of new articles published in this area. In fact, approximately two-thirds of the clinical articles discussed in this review were published in the last 8 years. The range of tools and concepts available from statistical physics and complexity science with which to tackle these problems is staggeringly broad and continually expanding. Determining how these tools and concepts inter-relate and in which research situation to use them should be an ongoing focus of research. Insights from other sciences−including geophysics, finance, applied physics, and signal processing, where these ideas are also commonly circulated – will undoubtedly shape the future of this already multidisciplinary field.

The future of criticality in the clinical arena will depend in large part on the formation of multidisciplinary teams in which physicists, mathematicians, data scientists, and clinicians collaborate to better answer a clinical question through the lens of criticality. While none of the applications mentioned in this review has yet to become mainstream or routine, it is conceivable that, under the broad umbrella of quantitative analysis of EEG, MEG, and fMRI, several of these techniques may transition to the bedside if they prove helpful in the diagnosis, prognosis, or treatment of diseases. In addition to more detailed studies in the clinical areas mentioned in this review, one can expect the next decade to see innovative studies, anchored in criticality, in areas like addiction medicine, stroke, neuro-immunology, traumatic brain injury, and headache medicine.

There are inevitable hurdles when concepts from one field (i.e., statistical physics) are translated into another (i.e., biomedicine). Still, there can be no doubt that these hurdles are worthwhile if they open up new horizons for both the understanding and the treatment of brain-related diseases.

## Author Contributions

VZ evaluated the literature and wrote the manuscript.

## Conflict of Interest

The authors declare that the research was conducted in the absence of any commercial or financial relationships that could be construed as a potential conflict of interest.
